# Cyclic Fouling and Flux Recovery in a Piezoelectrically Actuated Polytetrafluoroethylene Membrane Module

**DOI:** 10.3390/membranes16100325

**Published:** 2026-09-29

**Authors:** Shuiyang Xu, Ning Qin, Yan Guo, Pan Zhao, Tianxin Li

**Affiliations:** 1 School of Energy and Environmental Engineering, University of Science and Technology Beijing, Beijing 100083, China; b20190075@xs.ustb.edu.cn (S.X.); qinning@ustb.edu.cn (N.Q.); ustbgy2015@163.com (Y.G.); 2 Tianjin College, University of Science and Technology Beijing, Tianjin 301830, China; 18811707205@163.com

**Keywords:** piezoelectric actuation membrane, fouling analysis and control, flux cyclic recovery, Hermia model, XDLVO

## Abstract

Membrane fouling limits pressure-driven filtration by increasing hydraulic resistance and cleaning demand. Here, an external lead zirconate titanate actuator (PZT) was coupled to a passive polytetrafluoroethylene (PTFE) microfiltration membrane to examine cyclic fouling and short-term flux recovery. The primary pressure series comprised chronological sodium-alginate filtration stages at 6–30 kPa using a nominal 5 μm membrane from two membrane histories; additional actuation-duration, dosing, imaging, and interfacial analyses were treated as separate evidence streams. Across 31 adjacent activation events, endpoint flux increased in 28 events and decreased in three, with a median change of 1351 L/m^2^h. These events were repeated observations within the two histories and did not constitute independent replicates or a matched sham control. Segment-wise Hermia fitting retained 39 decline windows, for which cake filtration gave the minimum Akaike information criterion (AIC) in 34 windows (87.2%); however, the selected model was recovered in fewer than 80% of bootstrap resamples for 15 windows, underscoring that the fits are empirical descriptors rather than unique mechanism identifiers. The measured real electrical input was about 1.5 W, whereas the estimated viscosity-related flux gain from the recorded temperature change was only 0.08–2.68%, indicating that bulk heating alone could not explain the observed recovery. Separate microscopy and extended Derjaguin–Landau–Verwey–Overbeek (XDLVO) analyses were consistent with removal or redistribution of surface deposits and altered post-treatment attachment propensity. Overall, the data establish a reproducible within-history temporal association between actuator operation and flux recovery, while the absence of a matched sham experiment precludes attribution to a single mechanical, hydraulic, electrical, or pressure-transient pathway.

## 1. Introduction

Pressure-driven membrane processes are central to desalination, water reuse and advanced water treatment because they combine high separation efficiency with modular operation and relatively low energy demand [1,2]. Their long-term performance, however, is often limited by membrane fouling, in which dissolved, colloidal and biological materials accumulate on the membrane surface or within membrane pores [3]. This accumulation causes flux decline, hydraulic-resistance growth and repeated cleaning requirements [4]. In organic-rich waters, natural organic matter, proteins [5], polysaccharides, colloids, microorganisms and extracellular polymeric substances can act together to produce pore blocking, cake-layer formation, adsorption-controlled irreversible fouling and biofouling [6]. Fouling is therefore not only a hydraulic transport problem, but also an interfacial physicochemical process governed by van der Waals [7], electrostatic and acid–base interactions [8].

Flux-decline models and resistance analysis provide useful tools for diagnosing membrane fouling, but they cannot resolve the dynamic behavior of actively cleaned membranes [9]. Classical Hermia-type laws and resistance-in-series analysis can identify complete blocking, standard blocking, intermediate blocking and cake filtration under simplified constant-pressure conditions [10]. In practical filtration, especially for mixed organic foulants, deposition [11], adsorption [12], cake compression [13], back transport and shear-induced removal can occur simultaneously [14]. The interpretation may become more complex when cleaning is applied intermittently, because the flux curve contains both fouling accumulation during filtration and partial foulant removal during activation. A single monotonic fouling model is therefore insufficient for describing systems in which fouling growth and cleaning-induced recovery alternate over repeated cycles [15].

Piezoelectric membranes offer a promising route to such active fouling regulation. By converting an applied electric field into mechanical deformation through the inverse piezoelectric effect, piezoelectric components can generate localized vibration at or near the membrane surface [16]. Compared with external ultrasound or bulk hydrodynamic disturbance, this design can deliver energy closer to the fouling interface, enable intermittent activation during filtration and potentially combine mechanical vibration with electric-field or piezo-catalytic effects [17]. These effects may enhance local shear, promote foulant detachment, weaken concentration polarization and alter electrostatic interactions. However, the dominant antifouling pathway is expected to depend on foulant chemistry, operating pressure and membrane structure, so performance data alone cannot explain why a given recovery response occurs.

Piezoelectric actuation can be implemented either by using a piezoelectric filtration material or by coupling a conventional membrane to an external actuator. These configurations should be distinguished because electrical excitation, membrane motion and contact with the feed differ. Kuscer et al. [18] integrated lead zirconate titanate actuators outside the filtration area of an alumina membrane and reported reduced humic-acid-induced flux decline. Chen and Pomalaza-Raez [19] showed that the antifouling performance of a piezoelectric PVDF membrane depended on the excitation frequency and operating mode; continuous vibration was effective under their kaolin-filtration conditions, whereas intermittent excitation did not show the same benefit. Pu et al. [20] demonstrated electrically driven oscillation of a ZnO-CNT/PVDF membrane and evaluated flux recovery after a defined filtration and water-cleaning sequence. Mao et al. [16] combined a piezoelectric quartz support with a superwetting separation layer for oil-in-water emulsions. We have compiled these research findings in Supplementary Table S1, which details the characteristic parameters of the piezoelectric films. These results support the feasibility of vibration-assisted fouling control, but their different foulants, membrane structures and recovery definitions preclude direct ranking using a single percentage.

Many reports emphasize individual pathways, such as vibration-induced detachment, electrostatic repulsion or catalytic degradation [4,21]. For mixed organic fouling, these pathways are coupled with hydrodynamic deposition, interfacial adsorption, EPS (extracellular polymeric substances)-matrix formation and irreversible pore constriction [6,22]. As a result, it remains difficult to connect macroscopic cyclic flux recovery with microscopic fouling-layer structure and membrane–foulant interaction energy. Existing studies on piezoelectric or vibration-assisted membranes have therefore left an important mechanistic gap. This gap limits the rational design of piezoelectric cleaning protocols, because a high transient flux recovery may reflect removal of a reversible surface layer, changes in pore accessibility, modification of interfacial adhesion, or a combination of these processes.

In this study, we designed a piezoelectric vibration-assisted polytetrafluoroethylene (PTFE) membrane module and used it to investigate cyclic mixed-organic fouling by sodium alginate (SA), humic acid (HA) and bovine serum albumin (BSA). We first quantified flux decline and piezoelectric recovery under different pressures, membrane pore sizes and foulant compositions. We then used segment-wise Hermia fitting and Hermia-cycle descriptors to separate post-recovery decline behavior from cross-cycle recovery and irreversible resistance accumulation. Finally, scanning electron microscopy (SEM), three-dimensional morphology, confocal laser scanning microscopy (CLSM)-polysaccharide/protein-rich organic fouling matrix analysis, colocalization analysis and extended Derjaguin–Landau–Verwey–Overbeek (XDLVO) calculations were integrated to connect flux recovery with fouling-layer disruption and interfacial adhesion. This combined framework clarifies how piezoelectric vibration regulates coupled fouling accumulation and removal, and provides mechanistic guidance for designing antifouling piezoelectric membrane systems.

## 2. Materials and Methods

### 2.1. Piezoelectric Membrane Filtration System

A piezoelectric vibration-assisted membrane filtration system was designed for constant-pressure cyclic filtration. The filtration module consisted of a piezoelectric ceramic (PZT-5H, Baoding Hongsheng Acoustic Electronic Equipment Co., Ltd. Baoding, China) actuator, a supporting vibration plate, a PTFE membrane (Haining Wanda Filtration Equipment Co., Ltd. Haining, China), a plastic mesh spacer and an acrylic housing. The membrane was mounted directly on the vibration plate so that mechanical oscillation generated by the PZT actuator could be transmitted to the membrane surface during filtration [18]. This configuration follows the logic of recent vibrating and piezoelectric membrane systems, in which localized oscillation [20] is used to intensify interfacial shear while avoiding the energy dissipation associated with bulk external sonication [23]. The feed solution was circulated by a centrifugal pump, and the retentate was returned to the feed tank for continuous operation. Permeate was collected through a conical channel and recorded in real time using an electronic balance.

The measurement and control system included pressure, temperature and weight sensors, a Modbus-RTU communication network, a pulse-width modulation (PWM) signal generator, a motor driver, a metal-oxide-semiconductor field-effect transistor (MOSFET) module and relay switches. A Python-based program (Python 3.8) recorded the signals and adjusted the pump speed to maintain the target transmembrane pressure (TMP). Maintaining a well-defined hydraulic condition is important because variations in flux or TMP can change the foulant transport rate toward the membrane and complicate the interpretation of intrinsic membrane–foulant interactions [24]. The PZT actuator was periodically powered by a high-frequency alternating-current source to generate cyclic vibration during filtration. The schematic diagram of the filtration and control system is shown in Figure 1, and a photograph of the experimental setup is provided in Figure S1.

PTFE membranes were purchased from Haining Wanda Filtration Equipment Co., Ltd. (Haining, China). Unless otherwise stated, membranes with a nominal pore size of 2 μm, thickness of 200 μm and porosity of 75% were used. Additional membranes with nominal pore sizes of 1 and 5 μm were used for the pore-size comparison. SA, HA, BSA, calcium chloride (CaCl_2_), sodium hydroxide (NaOH) and hydrochloric acid (HCl) were purchased from Sinopharm Medicine Holding Co., Ltd. (Shanghai, China).

(1) Hydrodynamic environment and piezoelectric response testing

The near-membrane hydraulic environment was estimated with a three-dimensional stationary COMSOL Multiphysics 6.0 (COMSOL Co., Ltd., Beijing, China) water-domain model based on the measured 142 mm membrane diameter, 6 mm channel height, and 6.35 mm tubing connections [25]. Measured pressure and cycle-averaged retentate/permeate flows were used to define the hydraulic boundaries. Membrane-surface velocity and wall shear-rate fields were extracted using a common surface mesh for the six pressure conditions. These quantities are model-derived estimates rather than direct shear measurements.

(2) Electrical characterization of the PZT actuation circuit

The PTFE membrane was a passive porous medium; mechanical excitation was supplied by an external PZT transducer coupled to the supporting plate. Terminal voltage and current were measured synchronously with an oscilloscope, high-voltage probe, and current transformer. RMS voltage/current, apparent power, and real power were calculated from paired waveforms. These values describe electrical input at the PZT terminals and are not interpreted as acoustic or mechanical power delivered to the liquid.

(3) Centre-point laser vibrometry and simulated vibration-field assessment

The vibration response of the supporting plate was characterized independently using a Polytec RSV-150 laser vibrometer (Polytec China Ltd., Beijing, China). The laser spot was positioned at the center of the supporting vibration plate, and the recorded velocity trace at 33.16 kHz was used as a center-point measurement of the plate motion [26]. The mean velocity was removed before estimating the RMS velocity. To express the result in displacement and acceleration units, an equivalent sinusoidal conversion was applied at the recorded operating frequency: vpeak = sqrt(2) × vrms, xpeak = vpeak/(2 × pi × f), and apeak = 2 × pi × f × vpeak. These values provide a center-point equivalent displacement and acceleration and were not interpreted as a full-field membrane displacement.

To complement the point vibrometry measurement, COMSOL simulation results were used to describe the spatial distribution of the vibrating support field. The simulated displacement field was normalized before comparison with the radial deposition pattern, and the simulation was used only as spatial evidence for non-uniform vibration distribution. The measured RSV-150 amplitude and the COMSOL spatial field were kept as separate evidence types unless explicitly normalized, because the former is an experimental point measurement while the latter is a model-derived field distribution. This distinction is consistent with previous studies showing that membrane vibration or piezoelectric actuation can mitigate fouling, while the local vibration amplitude, frequency, and hydrodynamic coupling must be specified for a given device geometry.

### 2.2. Preparation of Model Foulant Solutions

SA, HA and BSA were selected as representative polysaccharide, humic-substance and protein foulants, respectively. Polysaccharides can form hydrated gel-like layers [10], humic substances participate in electrostatic and calcium-mediated interactions [14], and proteins can adsorb within pores or near pore entrances depending on membrane–foulant affinity. Stock solutions of SA, HA, BSA and CaCl_2_ (1 g/L each) were prepared at least 24 h before use and stored in the dark at ambient temperature to allow hydration and equilibration. For the SA filtration experiments, 5 L of feed containing 0.1 g/L SA was prepared from the stock solution using background water with a conductivity below 20 μS/cm. The feed was continuously stirred and had a pH of approximately 7.3 ± 0.5 and a conductivity of 125 ± 3 μS/cm; its ionic strength was estimated to be approximately 0.85 mM. Additional stock solutions, where required by the experimental protocol, were introduced using a programmable dosing pump (Kamoer, KPK200, Kachuan’er Fluid Technology (Shanghai) Co., Ltd., Shanghai, China).

PTFE microfiltration membranes with nominal pore sizes of 1, 2, and 5 μm served as passive porous filtration media. The 5 μm membrane was selected as the principal condition because the study was designed to resolve cyclic resistance development and recovery in a relatively open microfiltration structure rather than to achieve molecular sieving of individual SA, HA, or BSA molecules. The Dynamic light scattering (DLS, Zetasizer Nano ZS90, Malvern Panalytical, Malvern, UK) measurements showed submicrometric apparent hydrodynamic populations, but these model organics are deformable macromolecular/colloidal species whose association state depends on solution chemistry. Published studies further show calcium-mediated association of alginate/humic substances and aggregation between BSA and humic acid. Consequently, nominal pore size and DLS size should not be interpreted as a rigid-particle sieving relationship. The 1 and 2 μm membranes were used only as pore-size comparison conditions, not as molecular-cutoff membranes.

### 2.3. Cyclic Filtration Experiments

The feed solution was circulated through the membrane module by a centrifugal pump under constant-pressure control. Permeate mass and retentate mass were recorded continuously using electronic balances. TMP was measured by pressure sensors and maintained using feedback control. The feed temperature was monitored throughout the experiment and maintained at 20 ± 1 °C using a positive temperature coefficient (PTC, Guangdong Fuxin Technology Co., Ltd., Foshan, China) cooling module. Constant-pressure operation was used here to observe spontaneous flux decline and recovery, whereas the real-time mass recording provided sufficient temporal resolution to separate decline segments from short piezoelectric recovery events.

The operating pressure ranged from 3 to 30 kPa, archived runs were acquired using a Python Control system. The duration comparison used sequential 120 and 240 s piezoelectric stages on the same 1 μm membrane and retained feed. The dosing comparison used a 3 μm membrane in an initially 5 L background-water system, followed by programmable HA, SA and CaCl_2_ additions. The successive-run comparison used three chronological records from the same 5 μm membrane history with 20 s actuation. These histories were analyzed separately from the primary six-pressure experiments. Background water was ultrafiltered water rather than analytical-grade ultrapure water, and the feed and collection vessels were exposed to ambient air. Background-feed operation was therefore not treated as a sterile or foulant-free control.

Permeate mass-flow rates were obtained from linear fits to rising mass segments between collection-cup drainage events. Fits were retained when R^2^ ≥ 0.98, at least three mass observations were available, and the calculated flow and pressure were positive. Only complete fitting segments contained within each analysis window were used. J_pre_ was the median flux within 15–2 min before piezoelectric onset, and J_post_ was the median within 1–15 min after piezoelectric ended. Relative recovery was defined as 100 × (J_post_/J_pre_ − 1).

Eligible event pairs required at least one complete fit in each window, no recorded fault, a change of no more than 10% between pre- and post-piezoelectric pressure medians, and at least 80% of raw pressure observations within ±10% of the pre-event reference. For inter-event decline, the interval began near the early post-piezoelectric peak, identified using a three-point running median within the first 8 min of the post-piezoelectric analysis interval. It ended before the next recorded dosing or piezoelectric event, with a 2 min exclusion margin, or at 58 min after the current piezoelectric onset or the selected stage endpoint, whichever occurred first. The starting and ending fluxes were the medians of up to three observations at the respective interval boundaries. Fractional loss was calculated as (J_start_ − J_end_)/J_start_ and averaged across available intervals. Non-declining intervals were retained. End-stage flux was the median of the final 5 min of available observations.

### 2.4. Flux and Resistance Calculation

The permeate mass was converted to permeate volume, and the instantaneous membrane flux was calculated as

(1) $$J=\frac{\Delta m}{\rho A\Delta t}$$

where *J* is the permeate flux (m/s), *m* is the permeate mass (g), ρ is the density of water (1 g/cm^3^), *A* is the effective membrane area (m^2^), and Δt is the filtration time (s). Filtration resistance was evaluated based on Darcy’s law in combination with the resistance-in-series model [12,15].

(2) $$J=\frac{\Delta P}{\mu Rt}$$

(3) $$R_{t}=R_{m}+R_{f}$$

(4) $$R_{f}=R_{r}+R_{ir}$$

where Δ*P* is the TMP (Pa), μ is the dynamic viscosity of water (Pa·s), *R_t_* is the total filtration resistance (m^−1^), *R_m_* is the intrinsic membrane resistance (m^−1^), *R_f_* is the total fouling resistance (m^−1^), *R_r_* is the reversible fouling resistance (m^−1^) and *R_ir_* is the irreversible fouling resistance (m^−1^). Reversible resistance refers to resistance associated with concentration polarization and loosely attached deposits that can be reduced by hydrodynamic or piezoelectric disturbance, whereas irreversible resistance refers to strongly attached foulants and pore-confined deposits that persist after cleaning [24]. This distinction is essential for cyclic filtration because a temporary flux jump after actuation does not necessarily imply removal of the irreversible resistance fraction.

For constant-pressure dead-end filtration, the Hermia model is usually expressed in terms of filtration time t and cumulative filtrate volume *V* [27]:

(5) $$\frac{d^{2}t}{dV^{2}}=K{\left(\frac{dt}{dV}\right)}^{n}$$

where *K* is the blocking constant and *n* is the mechanism index. If the effective membrane area A remains constant, the permeate flux-based form is

(6) $$-\frac{dJ}{dt}=kJ^{3-n}$$

where *k* incorporates the membrane area and the original Hermia constant. The four classical Hermia mechanisms [15], their model equations and integrated flux forms are provided in Table S2. Because these laws can fit similar flux curves under different physical retention mechanisms, model selection was interpreted together with recovery behavior, resistance partitioning and morphological evidence rather than as stand-alone mechanistic proof.

### 2.5. Piecewise Mechanistic Model for Cyclic Fouling-Cleaning Behavior

The cyclic flux profile contains fouling accumulation during filtration and a short recovery event after Piezo-resonant actuator activation. Classical Hermia models provide empirical descriptors of individual constant-pressure decline segments but do not contain a vibration-cleaning term. Here, Hermia parameters were therefore treated as curve-shape descriptors rather than unique mechanism identifiers. Parameters extracted from each post-recovery decline segment were combined with cross-cycle recovery indicators to estimate the reversible fouling-growth coefficient, the vibration-cleaning coefficient, and the irreversible fouling-accumulation coefficient. Within a decline segment without actuation, the model assumes that resistance growth can be approximated by a constant-pressure Hermia-type decay. The rapid increase in flux following actuation changes the initial resistance state assigned to the next segment. This temporal pattern is compatible with foulant-removal mechanisms reported for vibration-assisted and electric or acoustic field-assisted membrane systems [28].

Each activation event lasted only several tens of seconds, which was brief relative to the total filtration period of several tens of hours. Filtration between activation events was conducted without piezoelectric excitation and therefore followed the same operating principle as conventional pressure-driven filtration. Because the experiments were performed as continuous filtration runs, no independently matched sham-actuation control was included. Instead, flux immediately before and after each activation event was compared on the same membrane, thereby reducing variability arising from differences in membrane specimens and preceding fouling histories. The recurrence of activation-associated flux recovery over multiple cycles demonstrates repeatability within the recorded operating histories. The post-recovery starting flux, recovery amplitude, and cross-cycle trends in Hermia parameters were therefore used to describe the combined cyclic response.

(7) $$\frac{dR_{r}}{dt}=k_{f}-k_{d}g\left(t\right)R_{r}$$

(8) $$\frac{dR_{ir}}{dt}=k_{ir}$$

Here, *k_f_* (m^−1^min^−1^) represents the rate at which foulants generate reversible resistance on the membrane surface, *k_d_* (min^−1^) represents the removal capacity of piezoelectric activation toward the reversible fouling layer, and *k_ir_* (m^−1^min^−1^) represents the accumulation rate of irreversible fouling. The function g(t) describes periodic piezoelectric activation [10]:

(9) $$g\left(t\right)=\left\{\begin{array}{cc} 1-\exp\left(-\beta t_{\mathrm{mod}}/T_{clean}\right) & 0<t_{\mathrm{mod}}\leq T_{clean}\\ 0 & t_{\mathrm{mod}}>T_{clean} \end{array}\right.$$

where *t*_mod_ is the time within the current cycle and *T_clean_* is the duration of piezoelectric activation. During non-cleaning decline segments, *g*(*t*) = 0, and thus,

(10) $$\frac{d\left(R_{r}+R_{ir}\right)}{dt}=k_{f}+k_{ir}$$

Equation (10) indicates that the flux-decay rate within a single decline segment first reflects the total fouling growth rate. Disentangling *k_f_* and *k_ir_* therefore requires information from cross-cycle recovery and long-term flux loss.

For each flux curve, the local flux maximum after piezoelectric recovery was first identified in each 120 min cycle and used as the starting point of the subsequent decline segment. For the *i*-th decline segment, the local time and normalized flux were defined as follows:

(11) $$\tau =t-t_{i,0}$$

(12) $$F_{i}\left(\tau \right)=\frac{J_{i}\left(\tau \right)}{J_{i,start}}$$

The four classical Hermia models were fitted to each normalized decline segment. The best-supported model among the four candidates was selected using the Akaike information criterion (*AIC*) [29]:

(13) $$AIC=N\ln\left(\frac{SSE}{N}\right)+2p$$

where *N* is the number of data points, *SSE* is the residual sum of squares, and *p* is the number of fitted parameters. In parallel, an extended Hermia model with a continuous mechanism index *n* was fitted to obtain flexible decline-shape descriptors.

For each cyclic flux curve, Hermia-cycle descriptors were extracted from all valid decline segments. These descriptors included the mean classical Hermia coefficient ${\bar{k}}_{classic}$, the slope of the classical coefficient with cycle number, the mean fitted initial flux, the mean classical R^2^, the fraction of segments assigned to each classical Hermia mechanism, the mean extended Hermia mechanism index ${\bar{n}}_{ext}$, the slope of ${\bar{n}}_{ext}$, the mean extended Hermia coefficient ${\bar{k}}_{ext}$, the slope of $k_{ext}$, the mean extended-model R^2^, the mean post-recovery starting flux, the trend in post-recovery starting flux, the mean recovery jump, the final flux, the final reversible and irreversible resistances, the final irreversible-resistance fraction and the mean fractional flux loss during decline segments. The overbar denotes the average across decline segments, and the slope denotes the linear trend of a descriptor with cycle number. These descriptors were used to connect empirical Hermia decline behavior with the mechanistic parameters of the cyclic fouling-cleaning model.

### 2.6. Membrane and Fouling-Layer Characterization

The surface morphology of membranes was characterized by scanning electron microscopy (SEM; ZEISS Sigma 300, Carl Zeiss AG, Oberkochen, Germany) after carbon sputter coating. SEM images were analyzed to quantify projected open area, local aperture distribution and broad solid-domain fraction. For the broad-domain analysis, continuous solid regions were evaluated using local-width thresholds from 20 to 100 μm.

Three-dimensional surface topography was measured using a laser confocal microscope (VK-X3000, Keyence Corporation, Osaka, Japan). The acquired data were reconstructed using VK-H1XM-MultiFileAnalyzer software. Areal surface parameters, including arithmetic mean height Sa, maximum height Sz, texture aspect ratio Str, arithmetic mean peak curvature Spc and developed interfacial area ratio Sdr, were used to describe vertical roughness, surface directionality, peak geometry and developed surface area. Areal surface parameters defined according to ISO 25178 [30], including Sa, Sz, Sdr, Str and Spc, were used to describe surface-height variation, developed interfacial area, texture directionality and summit geometry. In addition, full height matrices were extracted where available for independent profile and height-span analysis. The Keyence measurements describe the surface topography of the selected fields and should not be interpreted as membrane-wide averages.

The hydrodynamic size distributions of BSA, SA and HA were measured by dynamic light scattering (DLS; Zetasizer Nano ZS90, Malvern Panalytical, Malvern, UK). Measurements were performed at 25 °C with a scattering angle of 90°, and each sample was measured three times. DLS intensity distributions were interpreted as scattering-weighted size distributions rather than particle-number or mass distributions.

### 2.7. Surface Interaction Analysis

Membrane and foulant surface properties were characterized by contact angle and zeta potential measurements. Contact angles were measured using a goniometer (DSA25, Krüss GmbH, Hamburg, Germany), and zeta potentials were measured using an electrokinetic analyzer (SurPASS 3, Anton Paar, Graz, Austria). Surface free-energy components were calculated using the van Oss–Chaudhury–Good approach [7]. Based on these parameters, the XDLVO theory was used to estimate interaction energies between foulants and membrane surfaces, including van der Waals, electrostatic double-layer and acid–base interactions [8]. The XDLVO analysis was included because organic fouling is often governed by coupled membrane–foulant and foulant–foulant interactions rather than by hydraulic transport alone [31]. For a detailed description of the XDLVO free energy calculation method, please refer to Section S1 in the Supplementary Materials.

### 2.8. Statistical and Image-Analysis Considerations

For model fitting, goodness of fit was evaluated using the coefficient of determination (R^2^) and residual patterns, whereas relative support among candidate models was assessed using the small-sample corrected Akaike information criterion (AICc) [32,33]. Samples used for scanning electron microscopy (SEM), Keyence three-dimensional surface-topography analysis, and confocal laser scanning microscopy (CLSM) were obtained from three membrane states: an unused pristine membrane (original), a membrane subjected to filtration without piezoelectric actuation ultrasonic cleaning (non-PUSC), and a membrane subjected to filtration with piezoelectric actuation ultrasonic cleaning (PUSC). Selected fields of view were analyzed for each imaging method.

## 3. Results and Discussion

### 3.1. Experimental Cyclic Fouling-Cleaning Behavior and Model Validation

(1) Flux performance of the piezoelectric membrane at different operating pressures

The experimental cyclic filtration tests first demonstrated the characteristic decline-recovery behavior of the piezoelectric membrane system under constant-pressure operation. Under all examined transmembrane pressures, the flux decreased progressively during filtration and increased rapidly after piezoelectric activation, forming a repeated fouling-cleaning pattern, shown in Figure S2. For detailed experimental data, including time-resolved curves of flux, pressure, and temperature, please refer to Figure S2 in the Supplementary Materials; for the segmentation of PZT piezoelectric vibration on/off intervals at six representative pressures, see Figure S3. This response differs from a conventional monotonic fouling curve because the piezoelectric event periodically changes the interfacial deposit state [18]. Similar flux-recovery behavior has been reported for ultrasonic and piezoelectric vibration-assisted membranes [34], where acoustic streaming [23], local shear and membrane oscillation can disturb weakly attached particles or cake layers [20]. Therefore, the present flux curves should be interpreted as coupled fouling-removal trajectories rather than as simple constant-pressure decline tests.

To examine the temporal relationship between actuator activation and flux recovery, 31 adjacent events were aligned to the inferred activation boundary and compared using the endpoint flux immediately before and after each event (Figure 2). Endpoint flux increased in 28 of the 31 events, whereas all three negative responses, which occurred within the 12 kPa history, were retained. The event-centered trajectories showed an abrupt increase in normalized flux after the boundary, followed by renewed decline during the subsequent filtration interval. These observations demonstrate a reproducible within-history temporal association between actuator activation and short-term flux recovery. However, the events are repeated observations nested within two membrane histories rather than independent experimental replicates, and the analysis does not constitute a matched sham control or isolate vibration from accompanying hydraulic, electrical, or pressure-transient effects.

(2) Calculation of fluid shear on the surface of a piezoelectric film

COMSOL estimates indicated a spatially heterogeneous near-wall hydraulic field, with elevated wall shear near the outer rim and lateral connection region (Figure 3). Thus, the lateral-feed geometry generated non-uniform local shear rather than a single uniform cross-flow condition over the membrane disc. The simulation is used here to define the hydraulic context of the experiments; detailed surface maps and numerical distributions are provided in the Supplementary Information Table S3.

(3) Electrical input and vibration level of the PZT-actuated support

Electrical characterization showed stable actuation near 33.16 kHz, as showed in Figure 4. Across five synchronized waveform pairs, the PZT terminal input was 872.0 ± 13.6 V RMS and 16.41 ± 0.71 mA RMS, corresponding to 14.32 ± 0.84 VA apparent power and 1.50 ± 0.02 W real electrical input. Centre-point laser vibrometry independently confirmed plate motion at the operating frequency. Full waveforms are provided in the Supplementary Information Figure S4. Thermal effects were too small to account for the observed flux recovery. Across the six pressure conditions, the event-window peak-temperature estimate corresponded to only 0.08–2.68% viscosity-related flux enhancement, substantially below the measured post-actuation recovery. The recovery is therefore attributed mainly to a reduction in apparent hydraulic resistance during PZT-driven vibration rather than to temperature-induced viscosity changes.

The COMSOL-based spatial analysis suggested that the PZT-induced vibration field should not be described as a uniform full-area membrane displacement (Figure 5). The simulated top-surface displacement showed a non-uniform annular pattern, with alternating high- and low-displacement bands across the radial direction. This spatial feature was broadly consistent with the image-derived radial deposition profile, which also varied across the membrane surface. Therefore, the RSV-150 result should be interpreted as a center-point experimental amplitude measurement, whereas the COMSOL result should be used as supporting evidence for spatially non-uniform vibration distribution. Together, these results indicate that PZT activation likely imposed localized high-frequency mechanical disturbances on the fouling layer. However, the photo-derived deposition index is semi-quantitative and the COMSOL field is simulation-based; thus, the comparison supports spatial consistency rather than proving direct causality or uniform full-surface membrane displacement.

### 3.2. Empirical Hermia Description of Flux Decline Across Pressure Levels

Segment-wise Hermia fitting was performed on the experimental flux records at 6, 9, 12, 18, 21, and 30 kPa (Figure 6). The analysis retained 39 selected decline windows, comprising 11, 6, 7, 5, 5, and 5 windows at the respective pressures. Each window was normalized by its starting flux and fitted independently with the four classical Hermia models [35]. The minimum Akaike information criterion (AIC) identified the best-fitting model [32], while residual diagnostics and bootstrap uncertainty estimates supplemented the goodness-of-fit assessment. Cake filtration provided the best empirical fit in 34 of 39 windows (87.2%). Its selection counts were 10/11, 5/6, 6/7, 4/5, 4/5, and 5/5 across the six pressure levels, respectively. The remaining windows favored complete blocking in one case, standard blocking in one case, and intermediate blocking in three cases. These results identify the Hermia equations that best describe the selected flux trajectories, rather than uniquely identifying their physical causes. In particular, the frequent selection of the cake-filtration model does not establish that cake growth was the sole or dominant resistance-generating process. Likewise, an intermediate-blocking fit does not independently demonstrate pore-mouth deposition or partial pore blockage. Combined fouling models explicitly accommodate contributions from pore blocking, pore constriction, and cake formation [36]. Similar trajectories can therefore be compatible with different processes or their combinations; fitting alone does not distinguish these physical explanations. The model assignments are therefore interpreted as empirical descriptions of the selected decline windows, not direct evidence of distinct physical fouling regimes.

Parameter uncertainty and model-selection stability were evaluated using 2000 circular-block residual bootstrap resamples per window, drawing on block-resampling methods for stationary dependent observations [37]. The resulting 95% confidence intervals for the normalized coefficient k and fitted initial normalized flux are reported in Table S4. Across pressure groups, the median relative confidence-interval width for k ranged from 5.0% to 18.1%. However, the originally selected model was recovered in fewer than 80% of resamples for 15 of the 39 windows. At 12 kPa, these frequencies ranged from 69.3% to 78.6%, compared with at least 99.3% at 18 and 21 kPa. Thus, relatively precise conditional parameter estimates do not necessarily imply a uniquely supported model assignment. These frequencies measure stability under resampling around the selected model, rather than the probability that a physical mechanism is correct. The fitted k values also depended on pressure, window, and model form, precluding a general claim that repeated activation consistently reduced fouling accumulation. Differences in normalized k alone cannot isolate cleaning-induced changes from the post-recovery state, normalization reference, or other factors affecting flux decline. The reported intervals remain conditional on the selected model, fixed window boundaries, and observed starting flux. They do not quantify variability among independent experiments or uncertainty arising from alternative segmentation. For detailed results, please refer to Table S4 in the Supplementary Material: Segment-wise Hermia parameter estimates, conditional bootstrap uncertainty, and model-selection stability for the six experimental pressure datasets. Accordingly, the Hermia results provide quantitative descriptors of flux decline that require complementary evidence for physical interpretation.

### 3.3. Effects of Operating Conditions and Foulant Properties on Cyclic Flux Recovery

Additional filtration records from the same apparatus were analyzed to examine how actuation duration and the evolving fouling state affected cyclic recovery. Three comparisons were selected: sequential 120 and 240 s piezoelectric actuation stages on a 1 μm membrane, successive dosing stages on a 3 μm membrane, and three successive runs using a 20 s piezoelectric stage on a 5 μm membrane. These records were separate from the six-pressure dataset. Figure 7 presents the measured trajectories and individual event responses; Table S5 summarizes recovery, inter-event decline and end-stage flux. Each trajectory was normalized by the flux before its first selected piezoelectric event, allowing stage-wise changes to be compared without equating the absolute permeabilities of different membranes.

Extending piezoelectric duration was associated with improved relative recovery and flux retention in the 1 μm membrane record (Figure 7a,b). The median post-piezoelectric increase rose from 4.98% at 120 s to 6.36% at 240 s, with four events in each stage. The mean fractional flux loss over the subsequent inter-event intervals decreased from 11.17% to 9.28%, while the end-stage normalized flux increased from 0.881 to 0.954. Thus, the longer-actuation stage showed both a larger relative recovery and less pronounced subsequent decline. However, the mean absolute mass-flow increase was 0.278 and 0.243 g/s, respectively. The improvement therefore concerned relative recovery and retention rather than a larger absolute increment. Because the 240 s stage followed the 120 s stage on the same membrane, the comparison describes a sequential operating response rather than an independently replicated duration optimum.

The dosing sequence produced a different relationship between relative recovery and absolute throughput (Figure 7c,d). The median recovery was 14.66% in the background stage (S0), 13.34% after HA addition (S1), 19.71% after subsequent SA and HA addition (S2), and 22.63% in the later SA/CaCl_2_-containing stage (S3), with two piezoelectric events per stage. In contrast, the mean absolute mass-flow increments decreased successively from 0.380 to 0.275, 0.227 and 0.196 g/s. The larger recovery percentages at later stages therefore did not signify greater recovered throughput; they also reflected lower pre-piezoelectric flux. S2 exhibited a mean inter-event loss of 18.02%, compared with 9.01% in S0, showing that appreciable recovery could coexist with rapid renewed decline. Studies of alginate and humic-acid fouling likewise distinguish fouling development from hydraulic reversibility [13]. Here, the observations support a dependence on the evolving organic-fouling background, without isolating a calcium-specific mechanism from the sequential additions and accumulated operating history.

Successive operation further showed that an unchanged actuation duration did not guarantee a constant recovery response (Figure 7e,f). For the three 5 μm membrane runs, each containing eight selected 20 s piezoelectric events, median recovery decreased from 9.21% to 1.18% and then 0.23%. Positive responses occurred in eight, eight and six events, respectively. The first run exhibited stepwise cumulative recovery, whereas the later runs showed an overall decline. The corresponding end-stage normalized fluxes were 1.854, 0.925 and 0.838. These observations associate the diminishing response with the evolving membrane/feed history rather than with a change in actuation duration alone. The interpretation is also consistent with the broader finding that ultrasonic cleaning depends on foulant characteristics [23], although that literature does not establish the specific cause of the present run-to-run differences.

Together, these comparisons show why cyclic cleaning should be evaluated using both recovery and subsequent loss. A large percentage increase after piezoelectric can coexist with a small absolute increment or rapid renewed decline, whereas repeated modest increases can sustain cumulative recovery. This distinction gives the piecewise fouling-cleaning description a practical role: it separates activation-associated recovery from the flux evolution between activations. The selected records provide within-history experimental trends, not matched independent sham controls or a factorial ranking of membrane pore size, composition and actuation conditions.

### 3.4. Hermia-Cycle Descriptors and Their Association with Mechanistic Fouling Parameters

The cyclic flux curves contain two distinct processes: monotonic flux decline during filtration and rapid flux recovery after piezoelectric activation. Because the recovery stage is not a Hermia-type fouling accumulation process, the complete cyclic curve should not be fitted as a single Hermia process. Instead, the local flux maximum after each piezoelectric recovery event was taken as the initial flux of an individual decline segment, and the subsequent monotonic decline segment was fitted independently. This segment-wise strategy allowed the fouling-accumulation stage within each cycle to be separated from the piezoelectric recovery stage, consistent with the caution that filtration laws alone cannot uniquely identify retention mechanisms without complementary process information [28,38]. However, the Hermia coefficient obtained from a single normalized decline segment cannot uniquely resolve all mechanistic parameters of the cyclic fouling-cleaning model. In particular, the decline rate within one cycle mainly reflects the combined resistance growth during filtration, whereas the separation of reversible fouling growth, piezoelectric cleaning and irreversible fouling accumulation requires cross-cycle information. Therefore, Hermia-cycle descriptors were further compared with mechanistic input parameters using Spearman rank correlation analysis [39].

To visually interpret the mechanistic meaning of the fouling-cleaning parameters and the extracted Hermia-cycle descriptors, a model-based sensitivity analysis was performed using the cyclic resistance-evolution framework (Figure 8). The total hydraulic resistance was expressed as the sum of intrinsic membrane resistance, reversible fouling resistance, and irreversible fouling resistance. The reversible resistance increased during filtration according to the reversible fouling growth coefficient *k_r_*, whereas its removal during the cleaning stage was controlled by the cleaning coefficient *k_d_*. The irreversible resistance accumulated according to the irreversible fouling growth coefficient *k_ir_*. The instantaneous membrane flux was then calculated from Darcy’s law as a function of the evolving total resistance. One-at-a-time parameter perturbations were conducted for *k_r_*, *k_d_*, and *k_ir_*, while two derived descriptors, *k_f_* = *k_r_* + *k_ir_* and *k_ir_*/*k_f_*, were further used to represent the total fouling intensity and the relative contribution of irreversible fouling, respectively. The resulting flux-time and resistance-time profiles were used only for mechanistic interpretation and descriptor visualization, rather than as independent experimental datasets.

Together, the Hermia and correlation analyses provide a bridge between empirical flux-decline fitting and the mechanistic fouling-cleaning model [2]. Segment-wise Hermia fitting identifies the dominant decline behavior within each cycle, while the descriptor-parameter association clarifies which mechanistic processes are represented by the extracted descriptors. As an auxiliary validation, inverse models were also evaluated to test whether the Hermia-cycle descriptors could recover the mechanistic parameters. The cross-validated model-performance metrics are summarized in Figures S5 and S6, and the corresponding feature-importance and prediction-performance plots are provided in Figure S7. These results are used only as supplementary evidence for descriptor identifiability, rather than as the main mechanistic basis of this study.

### 3.5. Multiscale Evolution and PUSC-Induced Disruption of the Mixed-Organic Fouling Layer

Representative DLS measurements provide size-scale context for the model foulants used in the filtration experiments (Figure 9a). BSA showed a broad multimodal intensity distribution, including a small population near 5.61 nm, intermediate populations at approximately 79–190 nm, and a dominant aggregate signal near 712 nm (intensity-derived D_50_ = 676.4 nm). HA exhibited a broad principal population near 295 nm (D_50_ = 268.4 nm), whereas SA showed populations near 91 and 396 nm (D_50_ = 320.5 nm). The small BSA population is consistent with literature values of approximately 7 nm for native BSA, whereas the much larger signals indicate aggregate populations rather than molecular dimensions [40]. This distinction is important because SA, HA, and BSA association states are solution-dependent: BSA–HA aggregation has been reported both with and without calcium, while SA–HA association is enhanced in the presence of calcium [41]. Calcium also changes alginate association through charge screening/bridging and can intensify organic fouling [42]. Therefore, the measured DLS distributions should be interpreted as apparent hydrodynamic populations under the present preparation conditions, not as fixed material constants. Although these dominant populations were below the nominal 2 μm membrane pore size, substantial fouling remains physically plausible through pore-mouth adsorption, internal deposition, deformable macromolecular transport, aggregate capture, and subsequent cake/gel-layer development [43]. Protein microfiltration studies have likewise demonstrated internal fouling even when protein dimensions are much smaller than membrane pores [44]. Because replicate preparation-, aging-, concentration-, and post-filtration retentate DLS datasets were not available, the present particle-size analysis is used for mechanistic context rather than for generalizing aggregation kinetics.

SEM observations showed how these dispersed foulants were reorganized into deposits substantially larger than the individual hydrodynamic species (Figure 9b–d). The original membrane exhibited an open, interconnected fibrous network, whereas the non-PUSC membrane contained film-like and perforated-sheet deposits spanning multiple fibers, obscuring pore openings and dividing the remaining voids into narrower passages. Across three fields acquired under matched conditions, the projected open area decreased from 56.52 ± 1.78% for the original membrane to 43.28 ± 5.04% for the non-PUSC membrane, a relative decrease of 23.4%. The area-weighted local-aperture D_50_ and D_90_ decreased from 4.65 ± 0.19 and 13.48 ± 0.57 μm to 3.92 ± 0.01 and 11.82 ± 0.30 μm, respectively. At the same time, broad solid domains with a local width of at least 20 μm increased from 0.72 ± 0.31% to 3.84 ± 2.03%, approximately 5.3-fold above the original-membrane value. The consistent separation of all original and non-PUSC fields supports widespread surface coverage rather than isolated pore plugging. Supplementary Figures S8 and S9 detail the quantitative analysis of membrane pore size based on SEM images and the sensitivity analysis. Similar surface-spanning deposits have been linked to cake formation and localized organic deposition in microfiltration studies, including high-frequency ultrasound observations of foulant distribution [45].

Keyence three-dimensional measurements independently confirmed the transition from lateral pore coverage to pronounced vertical heterogeneity as shown in Figure 10. The arithmetic mean height Sa increased from 7.044 μm for the original membrane to 39.119 μm for the non-PUSC membrane (+455.4%), while the maximum surface height Sz increased from 108.410 to 293.695 μm (+170.9%). The developed interfacial area ratio Sdr concurrently increased from 1.379 to 3.201 (+132.1%), showing that the fouled surface possessed a much larger developed area than its projected area. In addition, the texture aspect ratio Str decreased from 0.883 to 0.530, indicating a shift from a comparatively isotropic surface toward a more directionally heterogeneous texture [46], while the mean peak curvature Spc decreased from 1586.756 to 1095.281 mm^−1^, consistent with coating or blunting of the original sharper surface features by deposited material. Analysis of the full 1024 × 768 height matrices yielded Sa-like values of 7.113, 39.097 and 6.396 um for the original, non-PUSC and PUSC membranes, closely reproducing the software-derived Sa values and supporting the validity of the extracted CAG data. PUSC largely reversed these topographic changes [18]. Relative to the non-PUSC membrane, Sa, Sz and Sdr decreased by 83.8%, 64.1% and 67.5%, respectively, reaching 6.321 μm, 105.442 μm and 1.040 after cleaning. Str recovered to 0.719, and Spc returned to 1589.616 mm^−1^, nearly matching the original-membrane value. The independently extracted diagonal profiles showed the same tendency: after linear detrending, the central 5th–95th percentile height span increased from 38.607 μm in the original membrane to 168.271 μm in the non-PUSC membrane and then decreased to 23.734 μm after PUSC. These data demonstrate removal of the large-scale, spatially heterogeneous deposit and restoration of the membrane topography to a state close to the original surface. They do not, however, imply that every microscopic region was completely regenerated, because residual valleys and isolated deposits remained and the Keyence comparison represents one selected field per condition. Representative three-dimensional Keyence surface morphology maps are provided in Figure S10, which visually confirm the pronounced vertical heterogeneity of the non-PUSC membrane and the partial restoration of surface morphology after PUSC [47].

CLSM analysis was performed to examine the distribution and composition of the polysaccharide/protein-rich organic fouling matrix in the fouling layer [46]. The merged CLSM fluorescence images are provided in Figure S11, and the corresponding polysaccharide/protein-rich organic fouling matrix composition and JACoP-based spatial colocalization analysis [48] are summarized in Figure S12. Quantitative fluorescence analysis showed that polysaccharides were the dominant polysaccharide/protein-rich organic fouling matrix fraction in all samples, with alpha- and beta-polysaccharides together accounting for approximately 75.1–84.6% of the total fluorescence signal. Proteins and nucleic acids contributed lower fractions but may still participate in foulant adhesion and matrix cohesion. Compared with the non-PUSC membrane, the PUSC-cleaned membrane showed weakened colocalization among polysaccharide/protein-rich organic fouling matrix components, especially between nucleic acids and proteins, indicating that piezoelectric activation disrupted the spatial organization of the polysaccharide/protein-rich organic fouling matrix. These results suggest that PUSC mitigated membrane fouling not only by removing deposited foulants but also by weakening the structural integrity of the polysaccharide-rich polysaccharide/protein-rich organic fouling matrix network.

### 3.6. Interfacial Interaction Analysis Based on XDLVO Theory

To clarify the interfacial contribution to post-cleaning fouling behavior, XDLVO interaction energies were recalculated for SA, HA, and BSA against initially foulant-loaded, PUSC-treated, and matched non-actuated control membranes. The calculation incorporated contact-angle-derived surface-energy components, zeta potentials, and an ionic-strength scenario constrained by the measured conductivity of the 20 mg/L SA feed. Full equations, model parameters [7,8], ionic-strength sensitivity analysis, and distance-dependent interaction-energy profiles are provided in the Supplementary Information (Section S1). The condensed comparison in Table S7 shows that the PUSC-treated membrane retained a higher calculated attachment barrier and less favorable near-contact interaction energy than the non-actuated control for all three foulants. These results suggest reduced propensity for subsequent foulant re-attachment after PUSC treatment, although XDLVO analysis alone does not distinguish reduced deposition from vibration-induced detachment during cleaning.

The physicochemical surface properties used for XDLVO calculation, including contact angle-derived surface energy components and zeta potentials, are summarized in Table S6. The calculated interaction energy parameters for each membrane–foulant pair are listed in Table S7. The calculated surface energy components show that the interfacial behavior was strongly dependent on foulant chemistry [13]. SA and HA carried higher negative charges than BSA, whereas the membrane surfaces after treatment became weakly charged or nearly neutral, especially in the SA and HA systems [14]. This loss of charge contrast weakens long-range electrostatic repulsion and increases the likelihood that short-range LW and AB attractions dominate once foulants approach the membrane surface.

Figure 11 summarizes the calculated attachment barriers and near-contact total interaction energies for the 0.443 mM main scenario, with the ranges across the four ionic-strength scenarios indicated separately. The PUSC-treated membrane consistently showed a higher calculated attachment barrier than the non-actuated control for all three foulants. For SA, the barrier was 0.01080 mJ/m^2^ for the PUSC-treated membrane, whereas no positive barrier was identified for the non-actuated control. For HA, the corresponding values were 0.04272 and 0.00012 mJ/m^2^, respectively. For BSA, the PUSC-treated membrane also retained a modestly higher barrier than the non-actuated control, at 0.00419 versus 0.00325 mJ/m^2^. This PUSC-versus-control ordering remained unchanged throughout the 0.443–1.554 mM sensitivity range.

The near-contact total interaction energies were negative for all membrane–foulant pairs, indicating that short-range attraction remained thermodynamically favorable after the outer barrier was crossed. However, the PUSC-treated membrane showed less negative near-contact energies than the non-actuated control for SA, HA, and BSA. For example, the respective values for the PUSC-treated and non-actuated control membranes were −37.03 and −37.82 mJ/m^2^ for SA, −29.07 and −34.20 mJ/m^2^ for HA, and −63.43 and −63.85 mJ/m^2^ for BSA. No physically significant finite secondary minima were identified over the calculated separation range. These results suggest that PUSC modified the post-cleaning membrane surface toward a state that was less favorable for subsequent foulant re-attachment than the matched non-actuated control. This interpretation is consistent with the role of XDLVO interaction barriers in modulating initial attachment probability [4]. Nevertheless, the model does not independently distinguish reduced deposition from vibration-induced detachment during cleaning. Accordingly, the XDLVO analysis is used here as an interfacial explanation for the observed post-cleaning state, while the cleaning effect itself should be supported by the matched flux-recovery, resistance, and residual-fouling measurements.

## 4. Conclusions

This study establishes an evidence-calibrated description of cyclic fouling and activation-associated flux recovery in an externally actuated PTFE membrane module. In the primary six-pressure sodium-alginate dataset, 28 of 31 adjacent events showed positive endpoint flux changes, but the events were nested within two chronological membrane histories rather than independent pressure-treatment replicates. Among 39 selected post-recovery decline windows, cake filtration produced the minimum AIC in 34 windows, although bootstrap model-selection stability was below 80% for 15 windows. The Hermia assignments should therefore be interpreted as curve-shape descriptors and not as proof of a unique retention mechanism. Electrical and thermal measurements showed a real PZT input of 1.50 W and an estimated temperature–viscosity contribution of only 0.08–2.68%, so recorded bulk heating was insufficient to explain the recovery. Separate SEM, surface-topography, fluorescence-imaging, and XDLVO analyses were consistent with partial removal or redistribution of surface deposits and with changes in calculated post-treatment attachment propensity, but these comparisons remain limited by field-level sampling, sample-history uncertainty, and condition-specific model inputs. The present evidence supports a robust temporal association between actuator operation and short-term recovery, not exclusive causal attribution to vibration. Matched sham experiments, independent membrane replicates, time-resolved feed and retentate characterization, and fully documented surface-property measurements are required to quantify mechanism-specific effects and long-term energy efficiency.

## Figures and Tables

**Figure 1:**
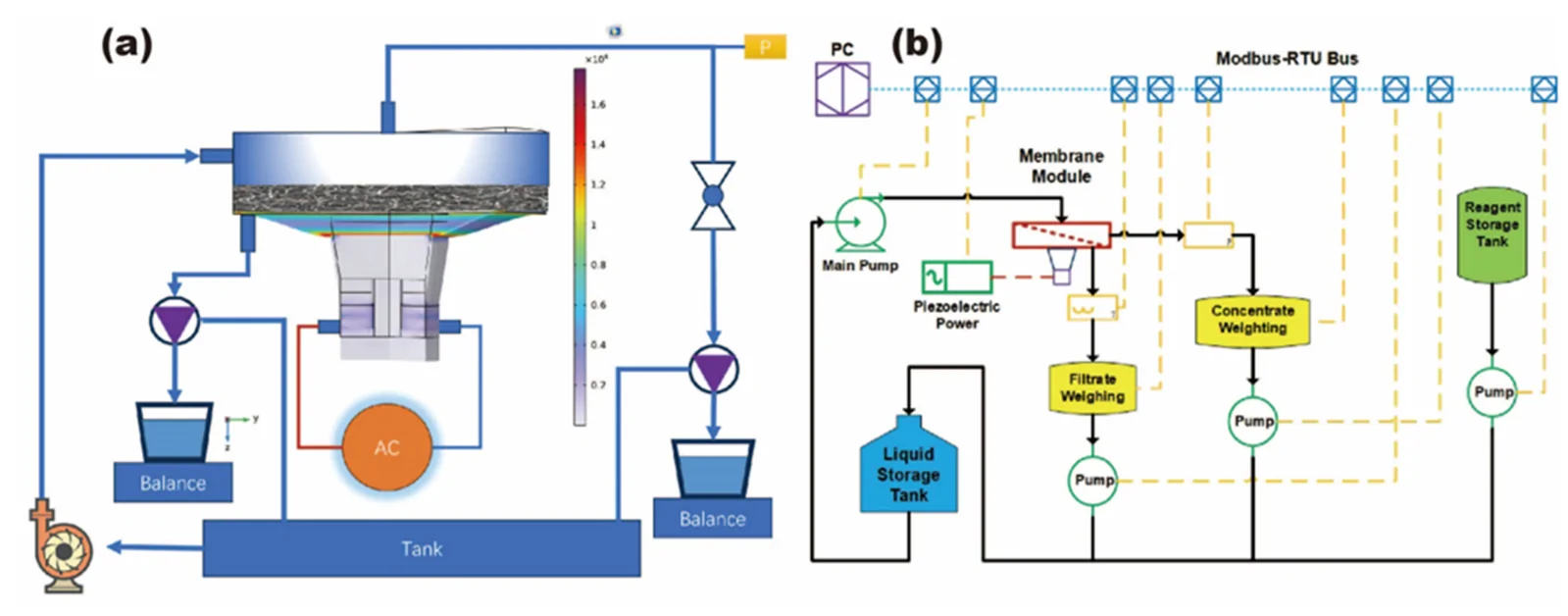
Self-assembled constant-pressure filtration system. (**a**) Schematic diagram of the system configuration. (**b**) Electrical measurement and control system.

**Figure 2:**
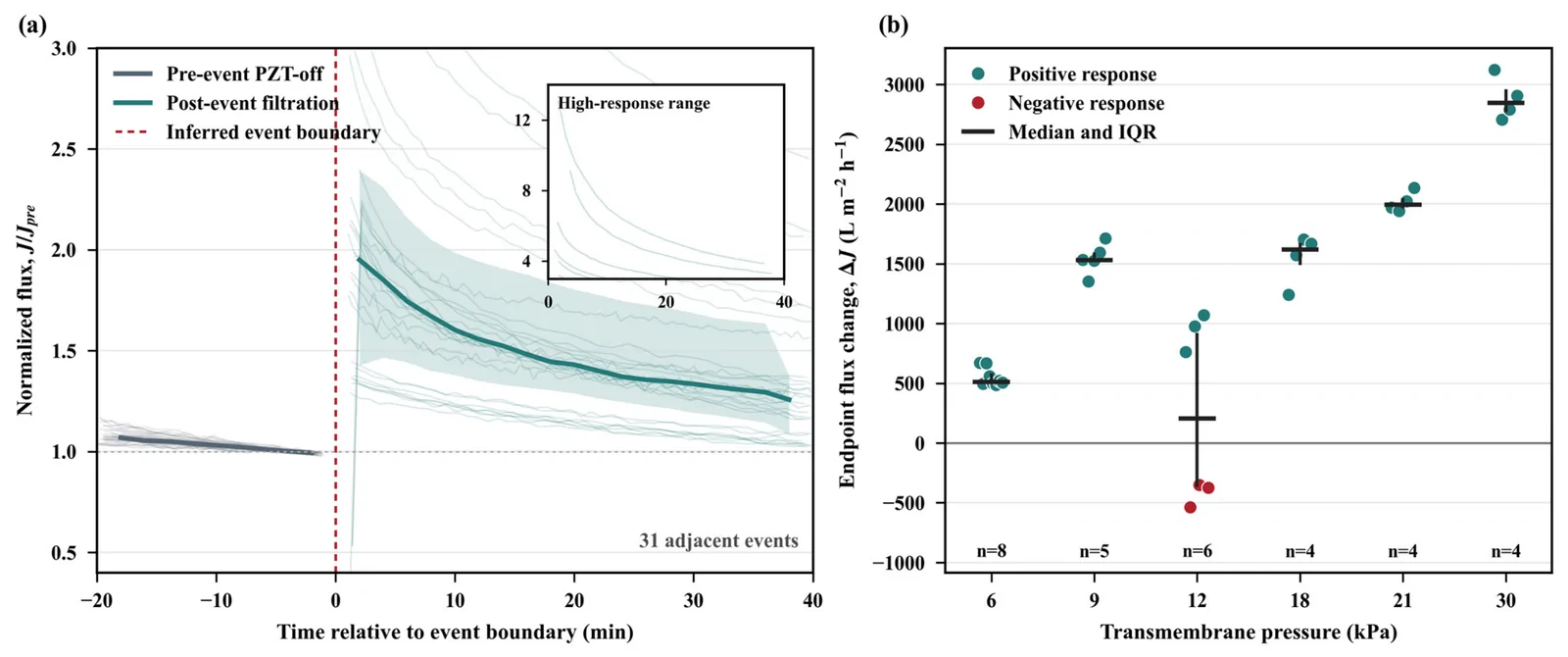
Event-centered flux response associated with PZT activation. (**a**) Thirty-one adjacent events aligned to the inferred activation boundary. Thin lines represent individual events; thick lines and shaded bands denote the median and interquartile range before and after the boundary. Flux is normalized to the median value during the final 5 min of the preceding PZT-off segment. The inset retains individual high-response trajectories above the main display range. (**b**) Endpoint flux change for every event at each pressure. Points show individual repeated events, horizontal bars show medians, and vertical bars show interquartile ranges. Positive responses occurred in 28 of 31 events; all three negative responses are retained. Events are repeated observations nested within two membrane histories and are not independent experimental replicates. No inferential between-pressure test was performed.

**Figure 3:**
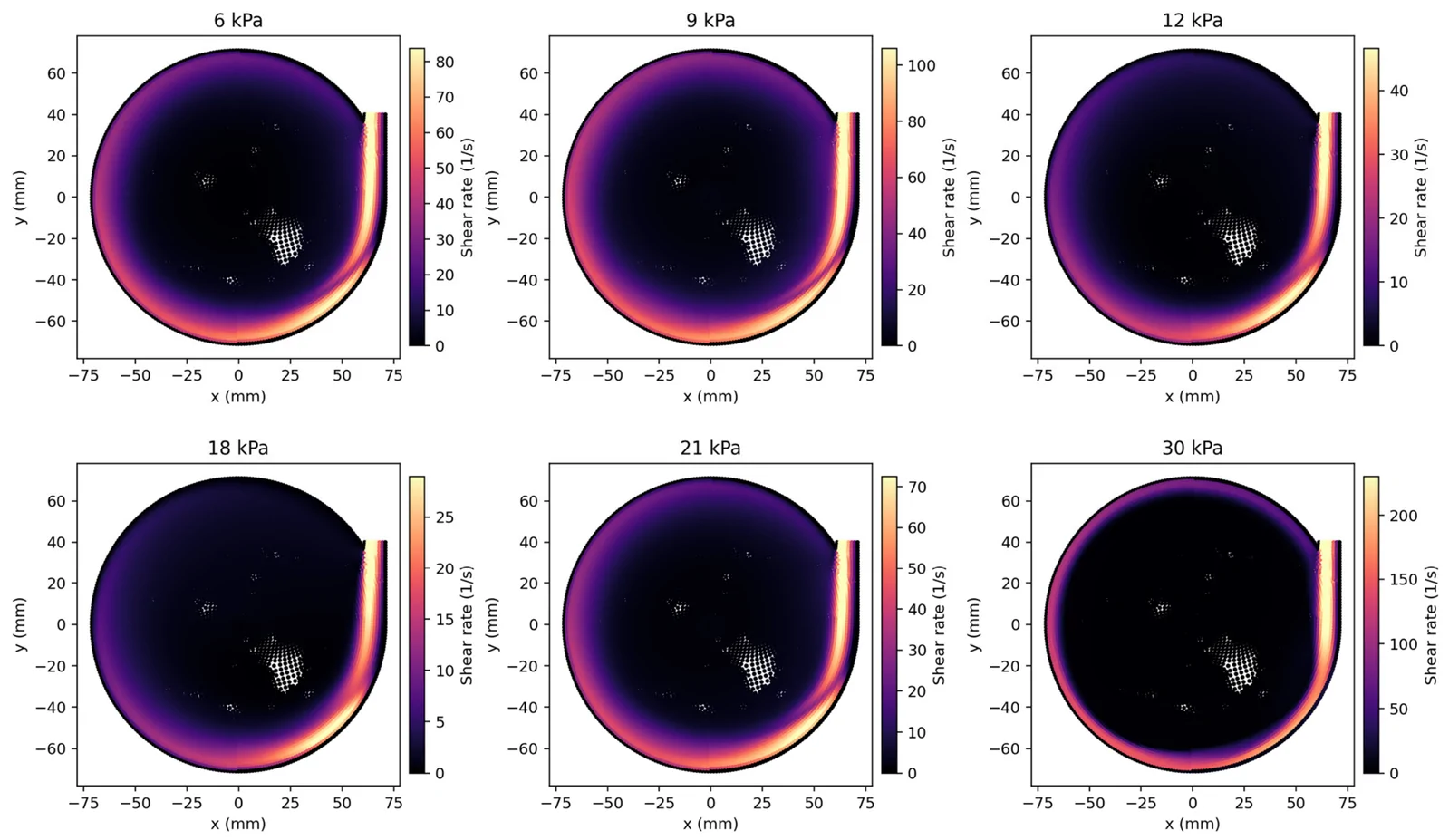
COMSOL-estimated membrane-surface wall shear-rate distributions under lateral-feed operation. Spatial maps of the model-derived wall shear rate on the membrane surface for the 6, 9, 12, 18, 21 and 30 kPa pressure cases. All maps were calculated from the stationary COMSOL water-domain model and plotted using matched membrane-surface coordinates.

**Figure 4:**
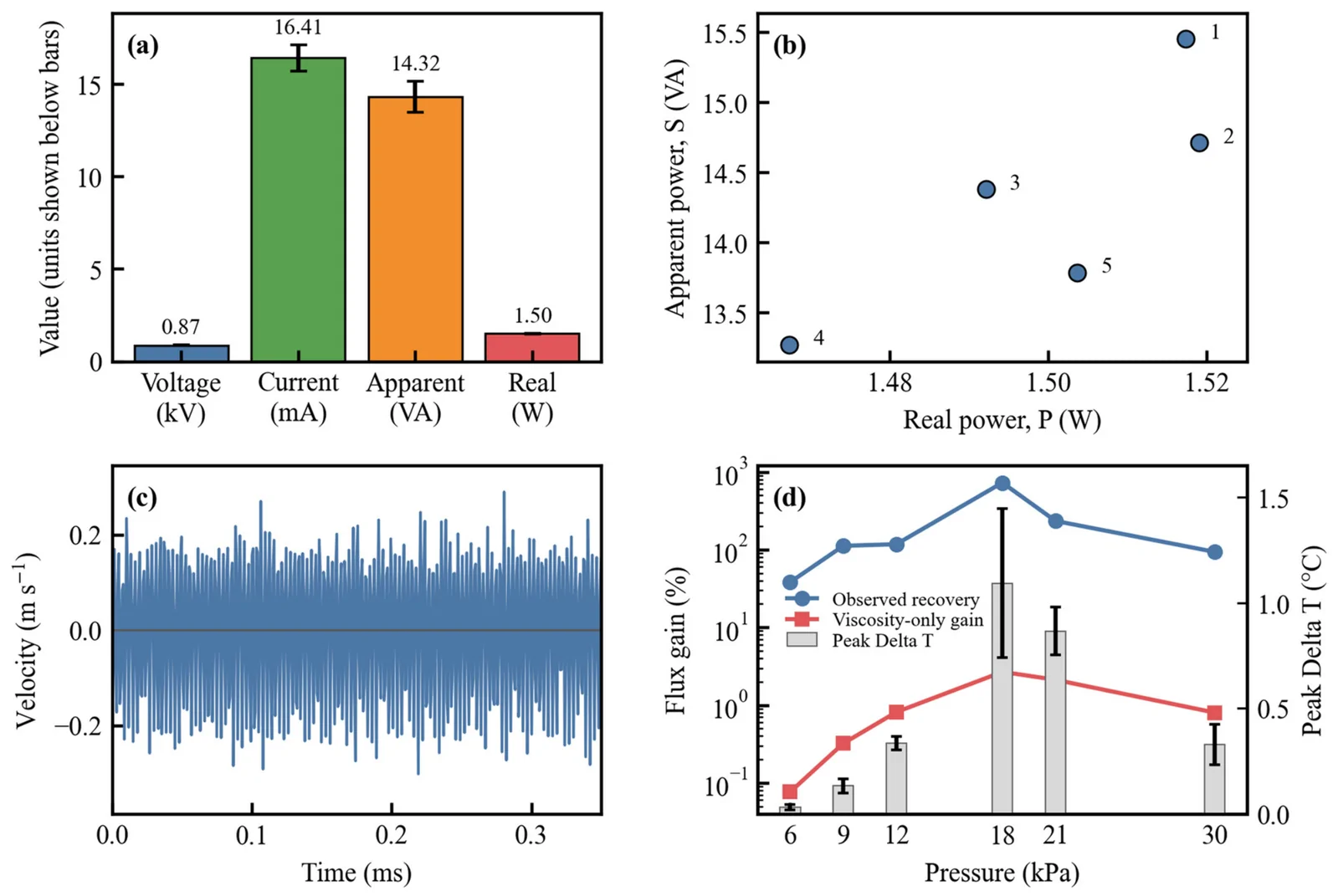
Electrical input, plate vibration, and temperature–viscosity contribution during PZT actuation. (**a**) RMS electrical input parameters at the PZT terminals, including voltage, current, apparent power, and real power. (**b**) Relationship between real power and apparent power calculated from five synchronized voltage-current waveform pairs. (**c**) Centre-point vibration velocity of the supporting plate measured by RSV-150 laser vibrometry. (**d**) Comparison of peak temperature rise, viscosity-only flux gain, and observed post-actuation flux recovery across the six pressure levels.

**Figure 5:**
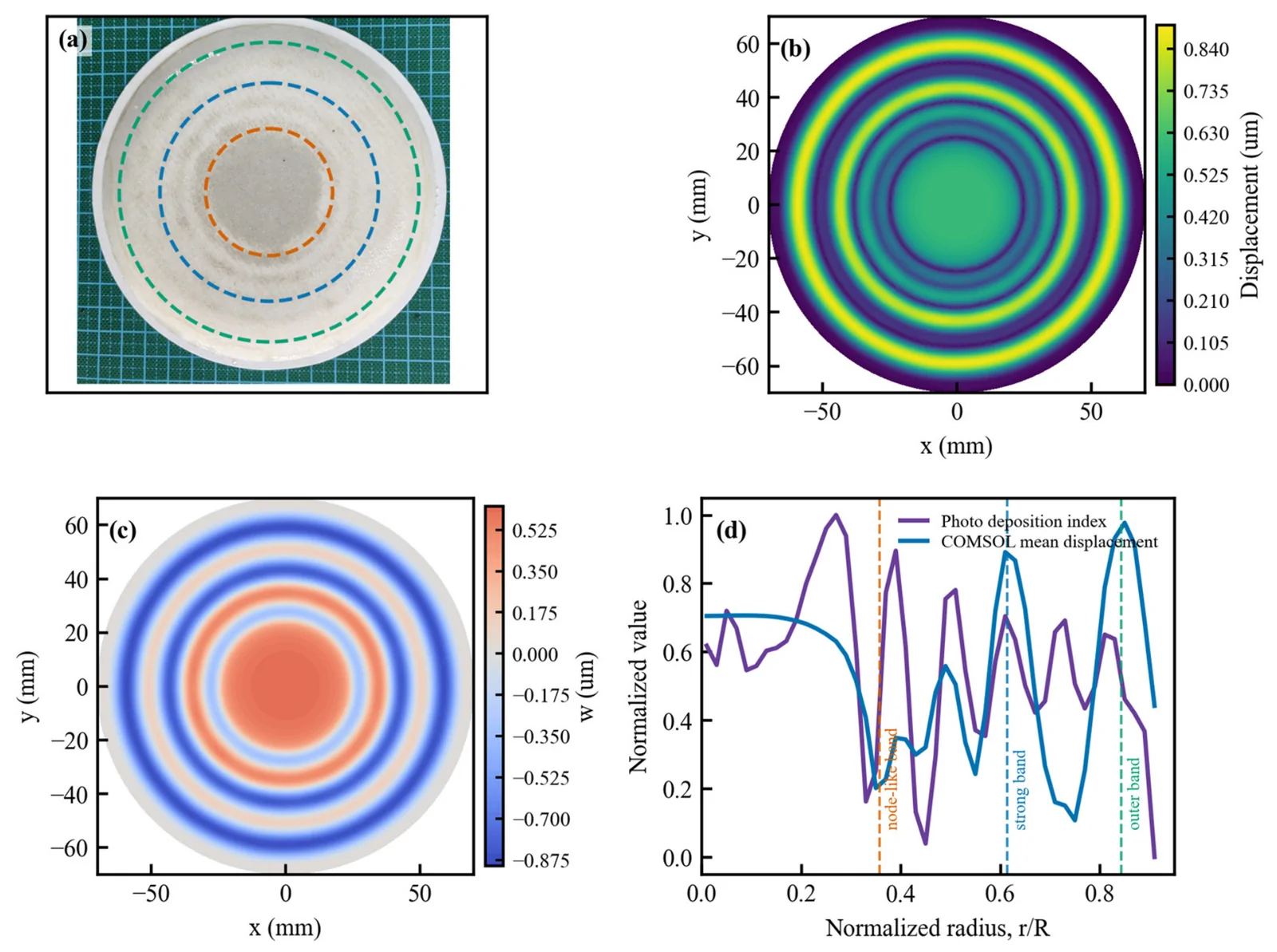
Spatial correspondence between visible fouling distribution and simulated vibrating-plate displacement. (**a**) Membrane photograph with radial ring overlays used for spatial comparison. (**b**) COMSOL-simulated top-surface displacement magnitude of the supporting plate. (**c**) COMSOL-simulated signed normal displacement component, showing the alternating annular bending pattern. (**d**) Normalized radial comparison between the image-derived deposition index and the simulated mean displacement profile.

**Figure 6:**
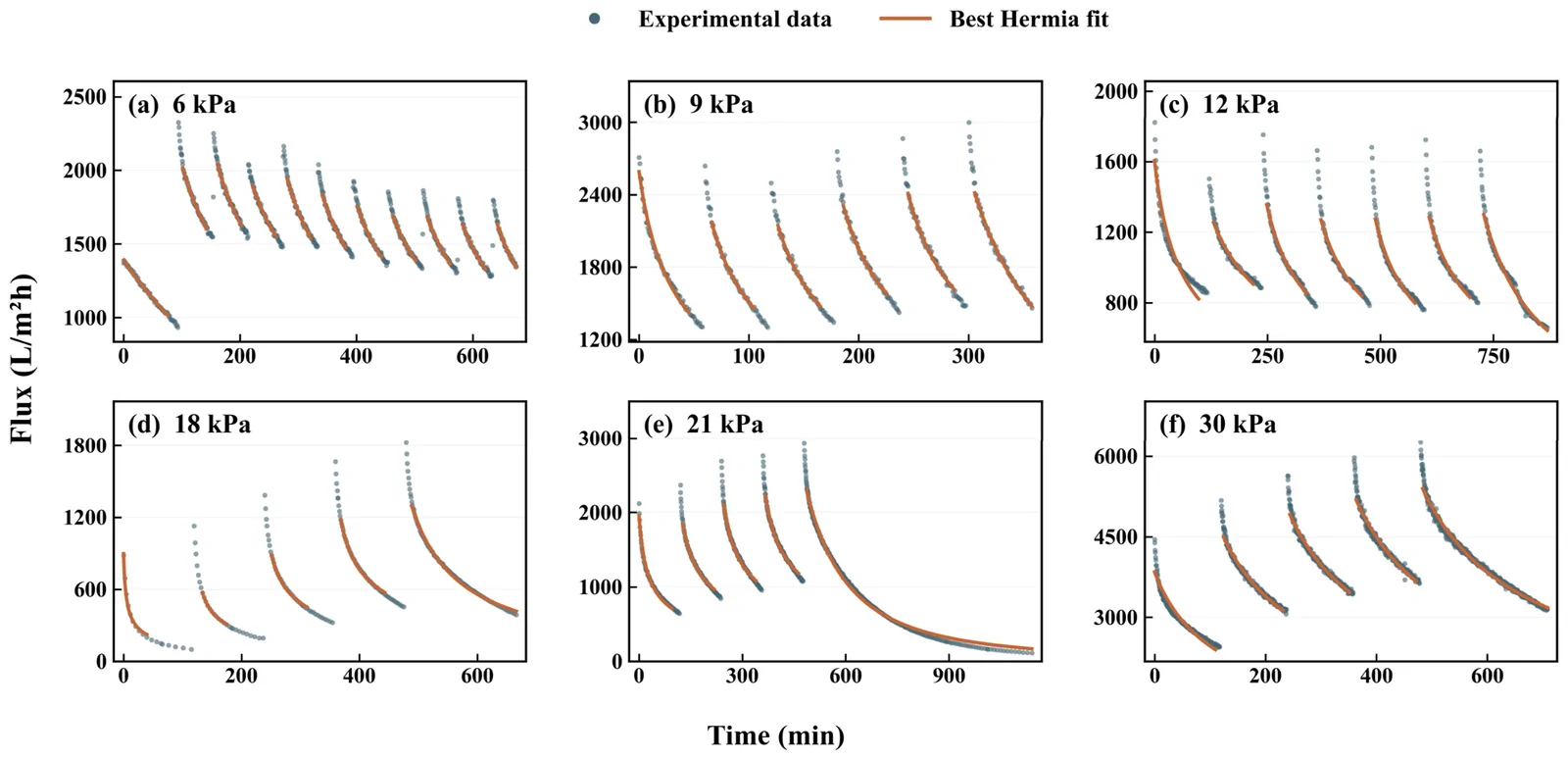
Segment-wise Hermia fits to experimental flux records at six filtration pressures. Panels show the records at (**a**) 6, (**b**) 9, (**c**) 12, (**d**) 18, (**e**) 21, and (**f**) 30 kPa. Blue-gray points represent all input observations, and orange lines represent the minimum-AIC Hermia fits within the selected decline windows. Each window was normalized by its starting flux for parameter estimation.

**Figure 7:**
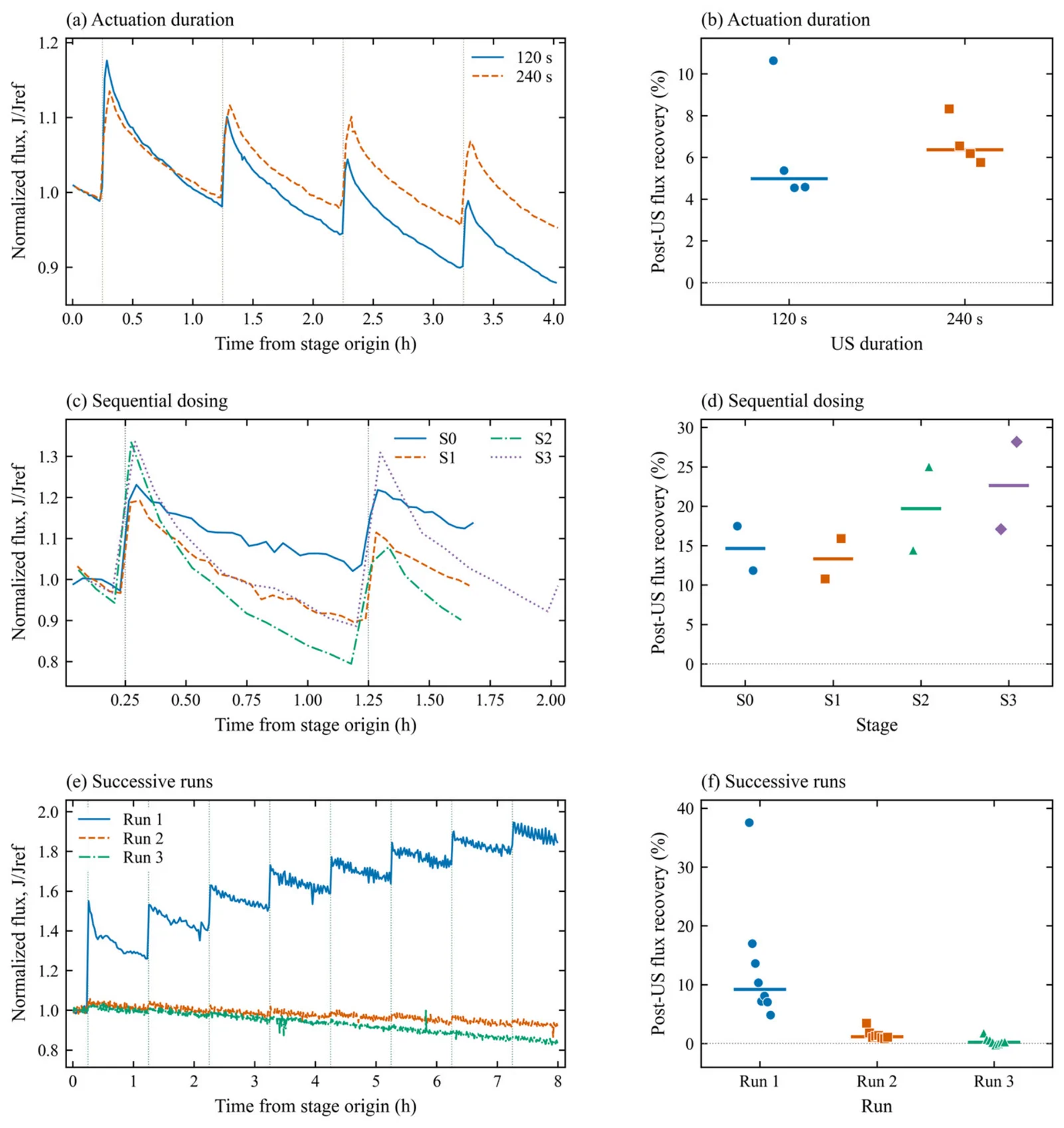
Measured cyclic flux responses to actuation duration, sequential dosing and successive operation. (**a**,**b**) A 1 μm membrane operated sequentially with 120 and 240 s piezoelectric actuation, with four selected events per stage. (**c**,**d**) A 3 μm membrane subjected to successive dosing: S0, background feed before the selected additions; S1, after HA addition; S2, after further SA and HA addition; and S3, after later SA/CaCl_2_ additions. Each dosing stage contains two events; the two S3 events have different cumulative calcium doses. (**e**,**f**) Three successive 5 μm membrane runs, each containing eight selected 20 s piezoelectric events. Left panels show balance-derived flux ratios J/Jref, where Jref is the median flux 15–2 min before the first selected piezoelectric event in each stage. Time zero is 15 min before that event. Dotted vertical lines mark recorded piezoelectric start times. Right panels show individual recovery values, calculated as 100 × (Jpost/Jpre − 1); horizontal bars indicate medians. Jpost is evaluated 1–15 min after piezoelectric ends. Horizontal point offsets improve visibility and have no quantitative meaning. Event counts represent repeated observations within membrane histories, not independent membrane replicates. Negative responses are retained. Curves are unsmoothed; stage durations differ. S3 has only one complete pre-piezoelectric mass-fit segment per event and is interpreted as lower-resolution supporting evidence.

**Figure 8:**
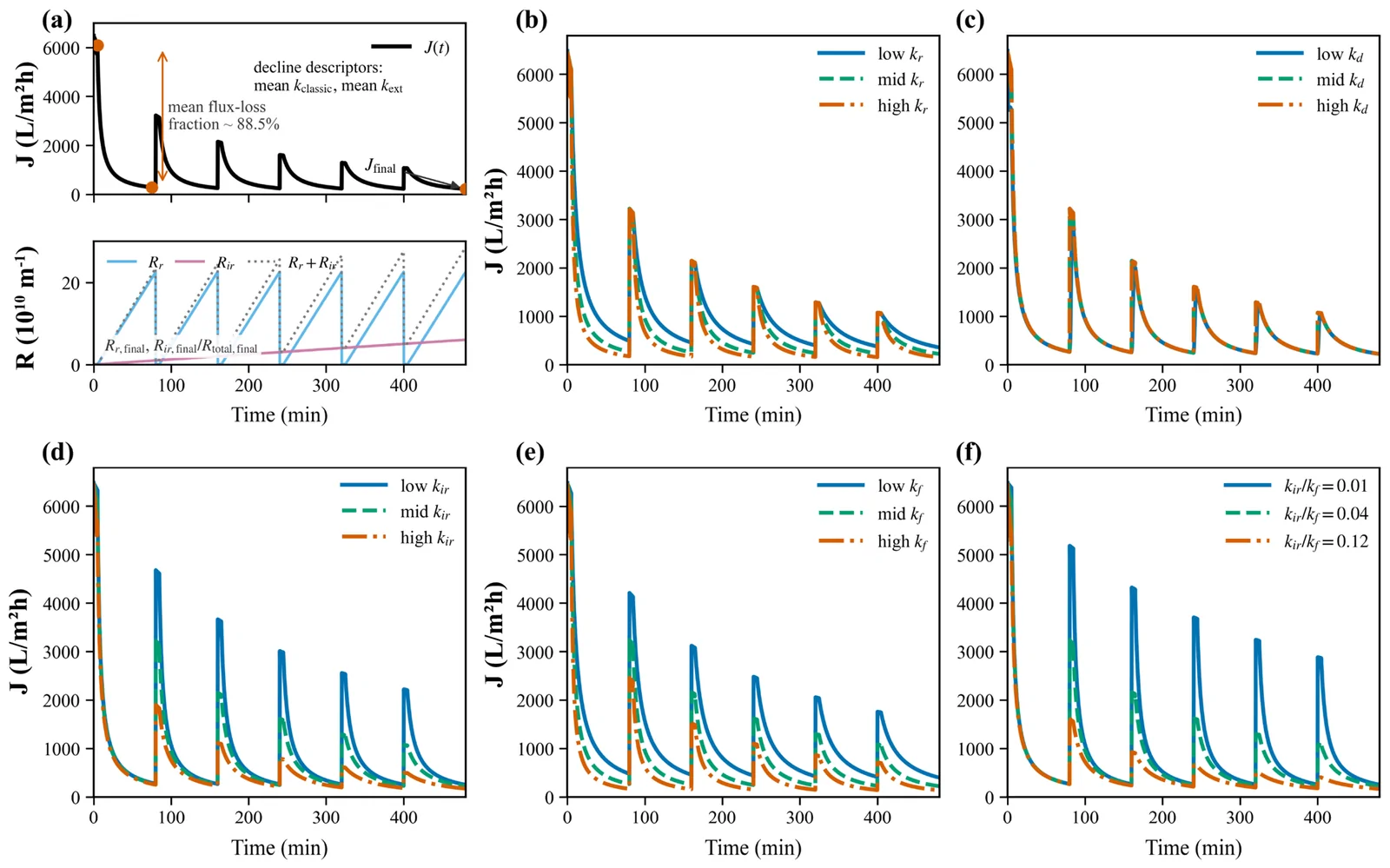
Model-based flux and resistance signatures of mechanistic fouling-cleaning variables. (**a**) Curve-derived response descriptors from a representative simulated cyclic flux and resistance profile. (**b**) Effect of reversible fouling growth coefficient *k_r_*. (**c**) Effect of cleaning coefficient *k_d_*. (**d**) Effect of irreversible fouling growth coefficient *k_ir_*. (**e**) Effect of total fouling growth coefficient *k_f_* = *k_r_* + *k_ir_*. (**f**) Effect of irreversible fouling fraction *k_ir_*/*k_f_*. The curves are model-generated and are intended to visualize descriptor meaning and parameter sensitivity rather than to represent independent experimental replicates.

**Figure 9:**
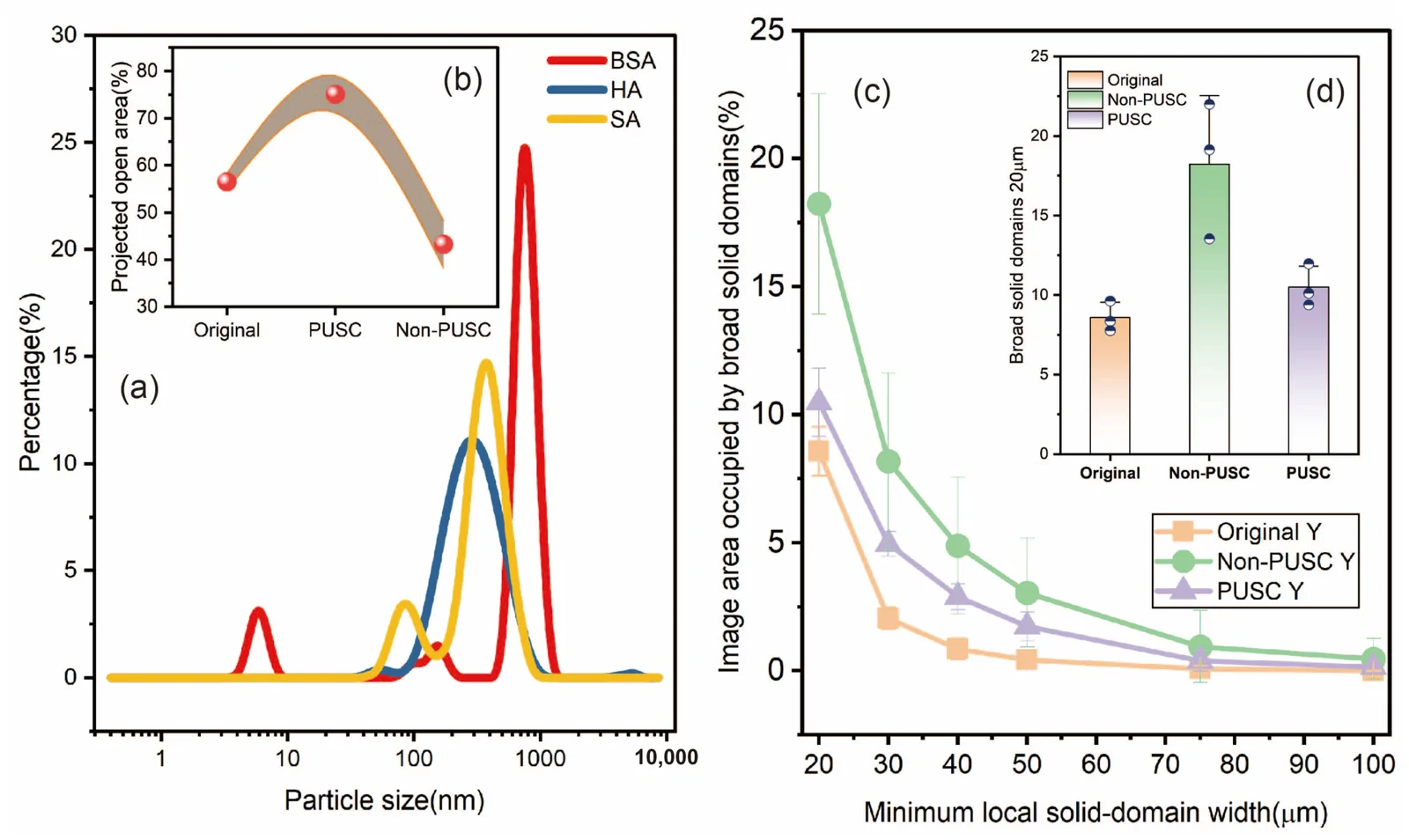
Particle-size distributions of the model foulants and SEM-based characterization of membrane surface morphology. (**a**) Hydrodynamic particle-size distributions of BSA, HA and SA. (**b**) Projected open area of the original, non-PUSC and PUSC membranes. (**c**) Image area occupied by broad solid domains as a function of the minimum local domain width. (**d**) Broad-solid-domain fraction at a minimum local width of 20 μm. Points represent individual SEM fields, and error bars denote field-level mean ± SD.

**Figure 10:**
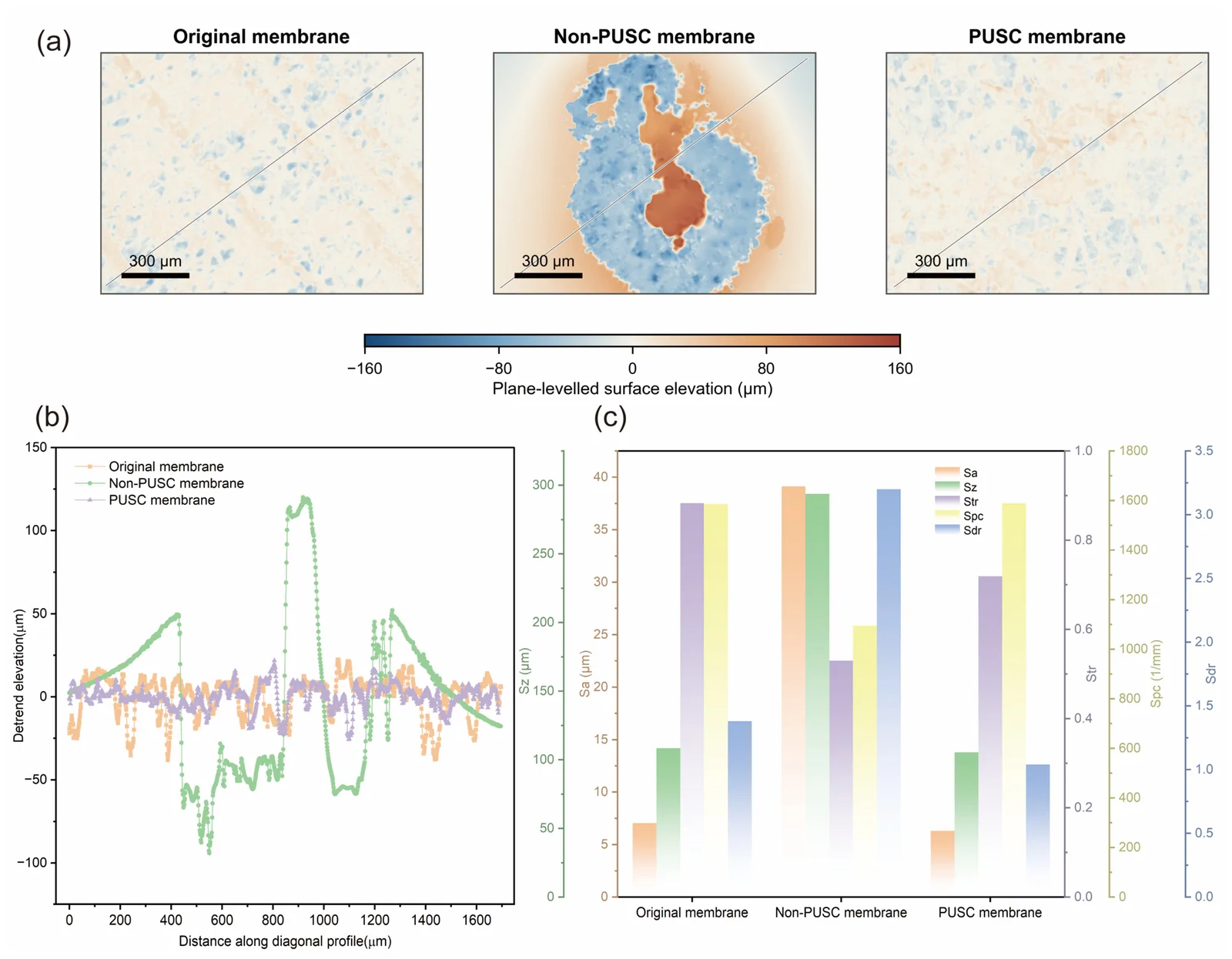
Surface topography, diagonal height profiles and areal surface parameters of the original, non-PUSC-fouled and PUSC-cleaned membranes. (**a**) Plane-levelled surface elevation maps reconstructed from the original Keyence CAG datasets. A common color scale (−160 to 160 µm) is used for all conditions, and the white/grey line marks the saved diagonal profile. (**b**) Linearly detrended elevation profiles along the selected diagonals. (**c**) Direct comparison of the Keyence areal surface parameters for the three membrane conditions, including arithmetic mean height (Sa), maximum height (Sz), texture aspect ratio (Str), arithmetic mean peak curvature (Spc) and developed interfacial area ratio (Sdr). Sa and Sz describe height amplitude, Str describes texture directionality, Spc characterizes peak geometry, and Sdr reflects the increase in developed surface area relative to the projected area. Each condition represents one Keyence field; the values are therefore interpreted descriptively without error bars or inferential statistics.

**Figure 11:**
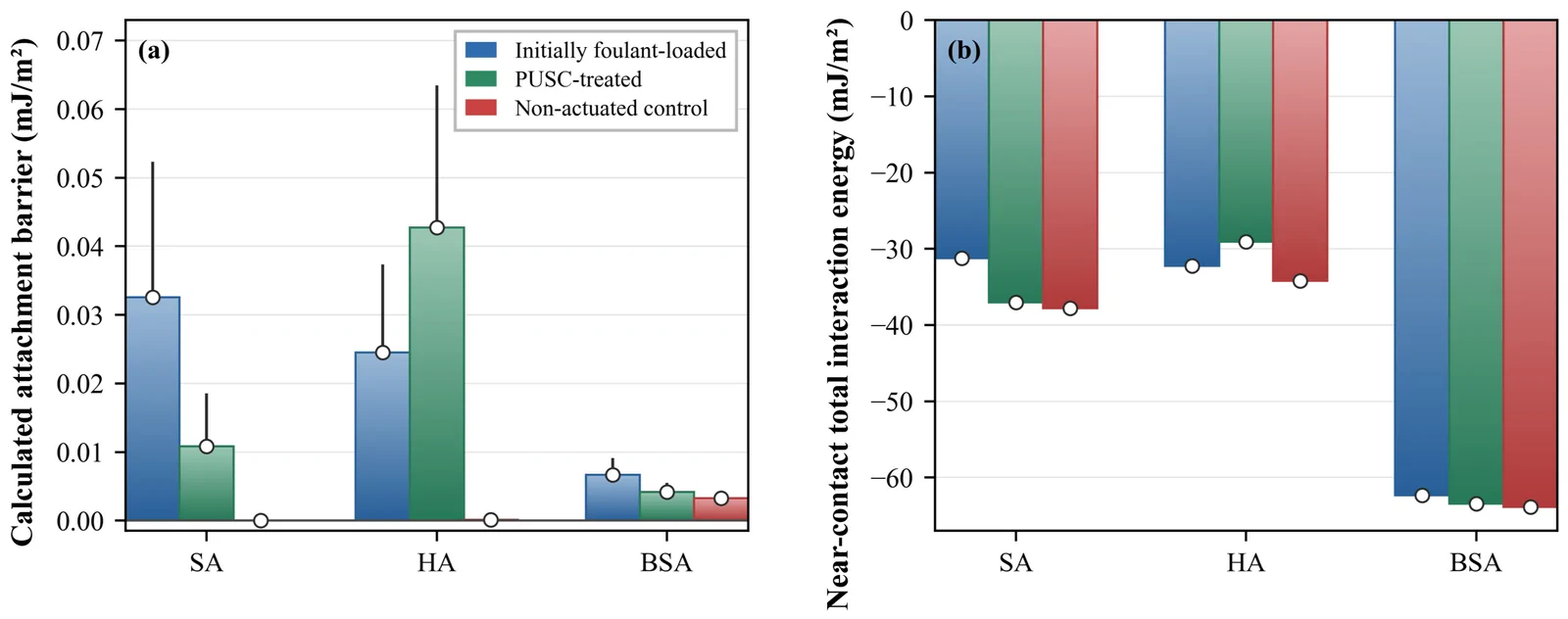
XDLVO-derived interaction metrics between foulants and membranes under different cleaning conditions. (**a**) Calculated attachment-energy barriers and (**b**) near-contact total interaction energies for SA, HA, and BSA on initially foulant-loaded, PUSC-treated, and non-actuated control membranes under the 20 mg/L SA feed scenario. Bars and white circles represent the main-case values; black vertical ranges indicate the ionic-strength sensitivity range.

## Data Availability

The raw data supporting the conclusions of this article will be made available by the corresponding author upon request.

## Supplementary Materials

The following supporting information can be downloaded at https://www.mdpi.com/article/10.3390/membranes16100325/s1: Table S1: Comparison of representative piezoelectrically actuated membrane systems with the externally actuated PTFE membrane module investigated in this study; Table S2: Four classical fouling mechanisms described by the Hermia model; Table S3: COMSOL-estimated membrane-surface velocity and wall shear-rate statistics under lateral-feed operation; Table S4: Segment-wise Hermia parameter estimates, conditional bootstrap uncertainty, and model-selection stability for the six experimental pressure datasets; Table S5. Descriptive metrics of the selected experimental stages; Table S6. Physicochemical surface properties of membranes and foulants, including contact angle-derived surface energy components and zeta potentials; Table S7. Calculated XDLVO interaction energy parameters for membrane–foulant pairs, including van der Waals, electrostatic, acid-base, and total interaction energies. Figure S1: Photograph of the experimental setup; Figure S2. Matched-pressure comparison of baseline flux decline and ultrasound-induced cyclic flux response across 3–30 kPa; Figure S3. Segmented cyclic flux profiles under six transmembrane pressures; Figure S4. Paired oscilloscope waveforms for PZT electrical characterization; Figure S5. Parameter-sweep simulation of Hermia-cycle descriptors under different mechanistic fouling-cleaning parameters; Figure S6. Inverse identification of piezoelectric membrane fouling parameters using Hermia-cycle descriptors; Figure S7: Spearman correlation and significance analysis of mechanistic fouling parameters and Hermia-cycle descriptors; Figure S8: SEM image segmentation workflow for projected open-void quantification; Figure S9: SEM-based projected open-area quantification and threshold-sensitivity analysis; Figure S10: Three-dimensional Keyence surface morphology of the original, non-PUSC-fouled, and PUSC-cleaned membranes; Figure S11: Merged CLSM fluorescence images showing the spatial distributions of α-polysaccharides, β-polysaccharides, proteins, and nucleic-acid-associated components in the pollutant mixture; Figure S12: EPS composition and spatial colocalization characteristics in the pollutant mixture and membrane samples.

## Abbreviations

The following abbreviations are used in this manuscript:

SA	sodium alginate
HA	humic acid
BSA	bovine serum albumin
XDLVO	extended Derjaguin–Landau–Verwey–Overbeek
CLSM	confocal laser scanning microscopy
PTFE	polytetrafluoroethylene
PZT	lead zirconate titanate
TMP	transmembrane pressure
