# Supplementary material for: Cyclic Fouling and Flux Recovery in a Piezoelectrically Actuated Polytetrafluoroethylene Membrane Module
Source: Membranes (Basel). 2026 Sep 29;16(10):325. doi: 10.3390/membranes16100325 (PMC13707183; doi:10.3390/membranes16100325)
Supplement: Supplementary file 1 [file membranes-16-00325-s001.zip › membranes-4528047-supplementary.pdf]

Cyclic Fouling and Flux Recovery in a Piezoelectrically Actuated Polytetrafluoroethylene Membrane Module

Shuiyang Xu[1,2], Ning Qin[1], Yan Guo[1], Pan Zhao[2], Tianxin Li[1*]

1 School of Energy and Environmental Engineering, University of Science and Technology Beijing, Beijing 100083, China

2 Tianjin College, University of Science and Technology Beijing, Tianjin 301830, China

* Corresponding authors: Tianxin Li

*E-mail: tianxinli@ustb.e*du.cn ;

Number SI pages: 19

Number the tables: 7

Number the figures: 12

This supporting information provides texts and figures addressing

Table S1: Comparison of representative piezoelectrically actuated membrane systems with the externally actuated PTFE membrane module investigated in this study.

Table S2: Four classical fouling mechanisms described by the Hermia model.

Table S3: COMSOL-estimated membrane-surface velocity and wall shear-rate statistics under lateral-feed operation.

Table S4: Segment-wise Hermia parameter estimates, conditional bootstrap uncertainty, and model-selection stability for the six experimental pressure datasets.

Table S5. Descriptive metrics of the selected experimental stages.

Table S6. Physicochemical surface properties of membranes and foulants, including contact angle-derived surface energy components and zeta potentials.

Table S7. Calculated XDLVO interaction energy parameters for membrane-foulant pairs, including van der Waals, electrostatic, acid-base, and total interaction energies.

Figure S1: Photograph of the experimental setup.

Figure S2. Matched-pressure comparison of baseline flux decline and ultrasound-induced cyclic flux response across 3–30 kPa.

Figure S3. Segmented cyclic flux profiles under six transmembrane pressures.

Figure S4. Paired oscilloscope waveforms for PZT electrical characterization.

Figure S5. Parameter-sweep simulation of Hermia-cycle descriptors under different mechanistic fouling-cleaning parameters.

Figure S6. Inverse identification of piezoelectric membrane fouling parameters using Hermia-cycle descriptors.

Figure S7: Spearman correlation and significance analysis of mechanistic fouling parameters and Hermia-cycle descriptors.

Figure S8: SEM image segmentation workflow for projected open-void quantification.

Figure S9: SEM-based projected open-area quantification and threshold-sensitivity analysis.

Figure S10: Three-dimensional Keyence surface morphology of the original, Non-PUSC-fouled, and PUSC-cleaned membranes.

Figure S11: Merged CLSM fluorescence images showing the spatial distributions of α-

polysaccharides, β-polysaccharides, proteins, and nucleic-acid-associated components in the pollutant mixture.

Figure S12: EPS composition and spatial colocalization characteristics in the pollutant mixture and membrane samples.

Table S1. Comparison of representative piezoelectrically actuated membrane systems with the externally actuated PTFE membrane module investigated in this study.

Study	Membrane and configuration	Feed and test context	Published actuation/performance	Insights from the literature
Kuscer et al., 2017	Conventional alumina membrane with external PZT elements outside the filtering area	Humic-acid filtration	100 kHz, 100 Vpp; 59% reduction in flux decline compared with no piezoelectric actuation	Supports external-actuator architecture; does not establish the present membrane's amplitude or efficacy
Chen and Pomalaza-Raez, 2019	Piezoelectric PVDF film, pin-punched holes; dead-end system	0.5 g/L kaolin; Z-average 907.7 nm; 34.5 kPa	Continuous sine excitation at 24 Vpp and 1601 Hz gave 87 ± 3% higher permeate flux than control; intermittent excitation did not show a comparable benefit	Shows mode/frequency dependence and cautions against assuming intermittent cleaning is universally optimal
Pu et al., 2021	ZnO-CNT/PVDF self-vibrating membrane	1000 ppm BSA in pH 7 PBS; other charged foulants also tested	8 kHz, 12.5 V AC; reported recovery 98.0% versus 58.5% unpowered composite and 53.0% driven bare PVDF	Recovery involved their defined filtration and water-cleaning sequence, including 20 min backwashing; not the same as our adjacent-cycle endpoint gain
Mao et al., 2023	Piezoelectric quartz support and attapulgite nanofibre layer; cross-flow emulsion filtration	500 ppm soybean-oil emulsion; 0.1 MPa; 1.5 m/s; 20 ± 3℃	Steady permeance 217 $L/m^2*h*bar$; approximately 25.3% increase with in situ ultrasound; rejection above 99.7%	Demonstrates combined wetting and actuation effects; does not validate organic-solute rejection or energy efficiency of our PTFE module

Study	Membrane and configuration	Feed and test context	Published actuation/performance	Insights from the literature
Present reanalysis	Passive nominal 5 µm PTFE with external actuator	Chronological SA pressure stages, 6–30 kPa; later mixed-organic evidence separately identified	28/31 positive endpoint events; median endpoint change 1351 L/m^2*h; no recovered independent sham control	Descriptive within-run recovery; no claim of superiority over the four published systems

Table S2. Four classical fouling mechanisms described by the Hermia model.

Mechanism	n	Differential form	Integrated flux form
Complete blocking	2	$-\frac{dJ}{dt}=kJ$	$J=J_0\exp(-kt)$
Standard blocking	1.5	$-\frac{dJ}{dt}=kJ^{1.5}$	$J=\left[J_0^{-0.5}+0.5kt\right]^{-2}$
Intermediate blocking	1	$-\frac{dJ}{dt}=kJ^2$	$J=\left[J_0^{-1}+kt\right]^{-1}$
Cake filtration	0	$-\frac{dJ}{dt}=kJ^3$	$J=\left[J_0^{-2}+2kt\right]^{-0.5}$

Table S3. COMSOL-estimated membrane-surface velocity and wall shear-rate statistics under lateral-feed operation. Statistics were calculated from the matched 11058-node membrane-surface export for each pressure case. Velocity values are reported as membrane-surface velocity magnitude, and shear-rate values are model-derived near-wall hydraulic estimates.

Pressure (kPa)	Velocity median (mm/s)	Shear-rate median (1/s)	Shear-rate P95 (1/s)	Shear-rate maximum (1/s)
6	0.459	6.53	55.58	114.11
9	0.534	11.7	74.14	139.11
12	0.293	2.77	28.04	68.24
18	0.189	1.75	17.47	44.49
21	0.379	5.4	47.14	100.75
30	1.213	1.06	137.48	285.15

Table S4. Segment-wise Hermia parameter estimates, conditional bootstrap uncertainty, and model-selection stability for the six experimental pressure datasets.

Pressure (kPa)	Window	Model	k (95% CI) (10^-3 min^-1)	J0/Jstart (95% CI)	R2	Selection frequency (%)
6	1	CB	4.131 (4.069-4.193)	1.0140 (1.0115-1.0166)	0.9958	90.5
6	2	CF	7.149 (6.989-7.314)	0.9874 (0.9844-0.9904)	0.9932	89.2
6	3	CF	7.111 (6.827-7.406)	0.9886 (0.9834-0.9936)	0.9876	76.1
6	4	CF	6.235 (6.031-6.428)	0.9898 (0.9861-0.9932)	0.9889	81.0
6	5	CF	7.417 (7.140-7.679)	0.9900 (0.9847-0.9947)	0.9910	80.4
6	6	CF	6.981 (6.640-7.311)	0.9893 (0.9834-0.9954)	0.9870	71.3
6	7	CF	6.579 (6.237-6.921)	0.9968 (0.9899-1.0032)	0.9842	69.9
6	8	CF	6.367 (6.052-6.682)	0.9905 (0.9851-0.9963)	0.9852	70.0
6	9	CF	6.781 (6.458-7.104)	0.9872 (0.9817-0.9932)	0.9880	71.7
6	10	CF	6.511 (6.340-6.683)	0.9962 (0.9929-0.9990)	0.9899	83.9
6	11	CF	7.595 (7.226-7.946)	0.9877 (0.9828-0.9933)	0.9886	69.5
9	1	CF	27.916 (26.095-29.628)	0.9565 (0.9360-0.9754)	0.9880	91.4
9	2	CF	15.641 (15.377-15.901)	1.0002 (0.9959-1.0038)	0.9953	99.8
9	3	CF	14.852 (14.418-15.282)	0.9841 (0.9780-0.9901)	0.9911	95.8
9	4	CF	14.749 (14.413-15.095)	0.9877 (0.9827-0.9927)	0.9920	98.5
9	5	CF	15.351 (14.892-15.806)	0.9755 (0.9688-0.9822)	0.9895	96.2
9	6	IB	12.670 (12.356-12.978)	0.9666 (0.9605-0.9724)	0.9942	92.1

Values in parentheses are marginal 95% percentile confidence intervals from 2,000 circular-block residual bootstrap resamples per window. k is based on normalized flux and is displayed in units of 10^-3 min^-1; J0/Jstart is dimensionless. CB: complete blocking (n=2); SB: standard blocking (n=1.5); IB: intermediate blocking (n=1); CF: cake filtration (n=0). Selection frequency is the percentage of resamples in which the originally selected model had the minimum AIC; it is not a probability that a physical mechanism is correct. Intervals are conditional on the selected model, fixed windows and observed Jstart, and do not represent independent experimental repeatability.

Table S4 (continued).

Pressure (kPa)	Window	Model	k (95% CI) (10^-3 min^-1)	J0/Jstart (95% CI)	R2	Selection frequency (%)
12	1	CF	18.621 (15.567-22.009)	0.8809 (0.8305-0.9397)	0.9380	70.0
12	2	CF	5.605 (5.101-6.113)	0.9662 (0.9514-0.9837)	0.9742	69.3
12	3	CF	9.247 (8.578-9.879)	0.9651 (0.9475-0.9835)	0.9842	78.6
12	4	CF	7.783 (7.131-8.446)	0.9612 (0.9441-0.9823)	0.9790	74.7
12	5	CF	9.764 (8.861-10.629)	0.9510 (0.9298-0.9761)	0.9808	77.3
12	6	CF	8.616 (7.809-9.507)	0.9463 (0.9253-0.9703)	0.9769	74.7
12	7	IB	7.557 (7.085-8.044)	0.9604 (0.9423-0.9825)	0.9883	69.3
18	1	CF	185.327 (168.229-203.314)	1.0150 (0.9854-1.0541)	0.9945	100.0
18	2	CF	28.893 (28.087-29.801)	1.0051 (0.9962-1.0127)	0.9991	100.0
18	3	CF	23.294 (22.400-24.254)	1.0077 (0.9929-1.0203)	0.9975	99.5
18	4	CF	21.551 (21.032-22.067)	1.0028 (0.9931-1.0111)	0.9989	100.0
18	5	IB	12.112 (11.742-12.471)	0.9736 (0.9601-0.9860)	0.9974	99.9
21	1	CF	40.438 (37.866-43.169)	0.9220 (0.8893-0.9468)	0.9882	99.3
21	2	CF	14.728 (14.419-15.041)	0.9879 (0.9799-0.9949)	0.9977	100.0
21	3	CF	15.251 (14.866-15.628)	0.9818 (0.9728-0.9900)	0.9969	100.0
21	4	CF	13.446 (13.154-13.746)	0.9888 (0.9807-0.9955)	0.9971	100.0
21	5	SB	8.377 (7.989-8.797)	0.9562 (0.9321-0.9788)	0.9965	100.0
30	1	CF	9.588 (8.213-10.878)	0.8645 (0.8330-0.9016)	0.9312	64.6

Pressure (kPa)	Window	Model	k (95% CI) (10^-3 min^-1)	J0/Jstart (95% CI)	R2	Selection frequency (%)
30	2	CF	5.288 (5.014-5.549)	0.9719 (0.9619-0.9829)	0.9866	83.3
30	3	CF	4.930 (4.715-5.148)	0.9752 (0.9672-0.9849)	0.9859	85.6
30	4	CF	4.986 (4.685-5.287)	0.9598 (0.9487-0.9722)	0.9809	74.5
30	5	CF	4.839 (4.697-4.978)	0.9304 (0.9220-0.9391)	0.9909	99.5

Values in parentheses are marginal 95% percentile confidence intervals from 2,000 circular-block residual bootstrap resamples per window. k is based on normalized flux and is displayed in units of 10^-3 min^-1; J0/Jstart is dimensionless. CB: complete blocking (n=2); SB: standard blocking (n=1.5); IB: intermediate blocking (n=1); CF: cake filtration (n=0). Selection frequency is the percentage of resamples in which the originally selected model had the minimum AIC; it is not a probability that a physical mechanism is correct. Intervals are conditional on the selected model, fixed windows and observed Jstart, and do not represent independent experimental repeatability.

Table S5. Descriptive metrics of the selected experimental stages.

Stage	Piezoelectric events	Median recovery (%)	Mean cycle loss (%)	End-stage J/Jref
Actuation duration: 120 s	4	4.98	11.17	0.881
Actuation duration: 240 s	4	6.36	9.28	0.954
Sequential dosing: S0	2	14.66	9.01	1.127
Sequential dosing: S1	2	13.34	12.93	0.992
Sequential dosing: S2	2	19.71	18.02	0.916
Sequential dosing: S3	2	22.63	9.47	0.953
Successive runs: Run 1	8	9.21	5.72	1.854
Successive runs: Run 2	8	1.18	2.87	0.925
Successive runs: Run 3	8	0.23	3.49	0.838

Piezoelectric events are repeated observations within membrane histories, not independent experimental replicates. End-stage ratios use a separate pre-US reference for each stage. Cycle loss is averaged over the available post-Piezoelectric intervals; their durations are not identical. S3 pools two successive calcium-containing stages, not two replicates at one concentration.

Section S1. XDLVO model formulation, input parameters, and ionic-strength sensitivity analysis

To examine whether piezoelectric ultrasonic surface cleaning (PUSC) altered the post-cleaning propensity for foulant re-attachment, the interaction between each foulant and three membrane states was evaluated using the extended Derjaguin–Landau–Verwey–Overbeek (xDLVO) framework. The membrane states were defined as an initially foulant-loaded membrane, a PUSC-treated membrane, and a non-actuated control membrane operated for the same duration under the same hydraulic conditions. The xDLVO approach combines Lifshitz-van der Waals (LW), Lewis acid-base (AB), and electrostatic double-layer (EL) interactions:

$$\Delta G_{TOT}(h) = \Delta G_{LW}(h) + \Delta G_{AB}(h) + \Delta G_{EL}(h)$$

The surface-energy components of SA, HA, BSA, and the membrane samples were obtained from contact angles measured with water, glycerol, and diiodomethane using the van Oss-Chaudhury-Good approach. The liquid surface-tension components used at 25 °C were 72.8, 64.0, and 50.8 mJ/m^2 for water, glycerol, and diiodomethane, respectively. The corresponding LW, electron-acceptor, and electron-donor components were 21.8, 25.5, and 25.5 mJ/m^2 for water; 34.0, 3.92, and 57.4 mJ/m^2 for glycerol; and 50.8, 0, and 0 mJ/m^2 for diiodomethane. The LW and AB contributions were propagated from the contact distance (h_0=0.158) nm according to

$$\Delta G_{LW}(h) = \Delta G_{LW}(h_0)\left(\frac{h_0}{h}\right)^2$$

$$\Delta G_{AB}(h) = \Delta G_{AB}(h_0)\exp\left(\frac{h_0 - h}{0.6}\right)$$

where 0.6 nm is the assumed AB decay length. The EL term was calculated using the measured zeta potentials, a relative permittivity of water of 78.5 (T=298.15K), and a 1:1 electrolyte approximation. Thus, all calculated quantities represent interaction free energies per unit area, rather than particle-scale attachment or detachment energies.

The main ionic-strength scenario was constrained by the actual filtration preparation. A 20 mg/L SA feed was prepared by diluting 100 mL of a 1 g/L SA stock solution to 5 L with purified water of approximately 20 μS/cm, yielding a measured conductivity of approximately 50 μS/cm. Following the documented historical dosing calculation, this condition was represented by an ionic-strength scenario of 0.443 mM. Because conductivity does not uniquely determine ionic strength in a polyelectrolyte-containing solution, this value was treated as a condition-specific estimate rather than a direct ionic-strength measurement. Additional scenarios of 0.847, 1.150, and 1.554 mM were calculated to cover the documented 100 mg/L SA condition and timer-inclusive

historical upper-bound dosing scenarios. The calculated distance range was 0.158–100 nm. A finite secondary minimum was only reported when a post-barrier local minimum exceeded ($1*10^{-3}$) mJ/m^2 in magnitude.

Table S6. Physicochemical surface properties of membranes and foulants, including contact angle-derived surface energy components and zeta potentials.

foulant	sample	theta_w θ_w °	theta_g θ_g °	theta_d θ_d °	zeta_mV ζ / mV	LW γ^{LW}	plus γ^{+}	minus γ^{-}	AB γ^{AB}	total γ^{total}
SA	Pure foulant	63.30±5.70	42.50±2.15	31.50±2.32	-34.62±3.25	43.59	2.31	7.98	8.58	52.17
	Initial fouled membrane	46.80±4.66	57.10±4.89	31.90±2.13	-14.32±1.10	42.70	0.00	36.56	0.00	42.70
	PUSC membrane	49.20±3.49	52.30±4.20	32.50±2.18	-7.95±0.60	43.16	0.09	30.71	3.35	46.51
	No-PUSC membrane	56.30±4.76	65.40±6.53	31.50±3.00	3.14±0.20	41.30	0.00	25.93	0.00	41.30
HA	Pure foulant	39.70±3.95	17.50±1.66	33.60±2.72	-25.83±1.99	42.67	3.96	22.32	18.80	61.47
	Initial fouled membrane	32.60±2.09	37.20±3.09	35.30±2.82	-13.32±1.19	41.89	0.90	40.31	12.03	53.92
	PUSC membrane	24.70±2.24	35.60±3.29	36.20±1.75	-19.64±1.88	41.47	0.81	47.69	12.39	53.86
	No-PUSC membrane	35.90±3.42	36.90±2.92	28.90±2.54	-1.17±0.12	44.67	0.80	35.65	10.71	55.38
BSA	Pure foulant	66.90±4.90	47.20±4.51	34.20±2.49	-8.63±0.83	42.40	1.95	6.85	7.32	49.72
	Initial fouled membrane	80.30±7.80	39.80±3.08	38.90±2.58	-12.32±1.02	40.14	6.68	0.00	0.00	40.14
	PUSC membrane	84.20±7.01	37.20±3.28	35.30±3.44	-8.64±0.76	41.44	5.92	0.00	0.00	41.44
	No-PUSC membrane	85.50±5.31	35.60±3.51	33.40±3.07	-7.32±0.66	42.13	5.71	0.00	0.00	42.13

Table S7. Calculated XDLVO interaction energy parameters for membrane-foulant pairs, including van der Waals, electrostatic, acid-base, and total interaction energies.

foulant	membrane	$\Delta G_{h_0}^{LW} / \left(mJ / m^2 \right)$	$\Delta G_{h_0}^{AB} / \left(mJ / m^2 \right)$	$\Delta G_{h_0}^{EL} / \left(mJ / m^2 \right)$	$\Delta G_{h_0}^{TOT} / \left(mJ / m^2 \right)$	$\Delta G_{barrier} / \left(mJ / m^2 \right)$	$\Delta G_{\min} / \left(mJ / m^2 \right)$
SA	Initial fouled membrane	-7.213	-24.011	-0.004	-31.228	0.045	-31.228
	PUSC membrane	-7.347	-29.584	-0.122	-37.053	0.015	-37.053
	No-PUSC membrane	-6.795	-30.752	-0.375	-37.922	-0.006	-37.922
HA	Initial fouled membrane	-6.718	-25.552	0.029	-32.241	0.033	-32.241
	PUSC membrane	-6.596	-22.538	0.091	-29.044	0.056	-29.044
	No-PUSC membrane	-7.506	-26.583	-0.145	-34.234	0.000	-34.234
BSA	Initial fouled membrane	-6.140	-56.192	0.018	-62.315	0.008	-62.315
	PUSC membrane	-6.514	-56.922	0.015	-63.421	0.005	-63.421
	No-PUSC membrane	-6.712	-57.143	0.012	-63.843	0.004	-63.843

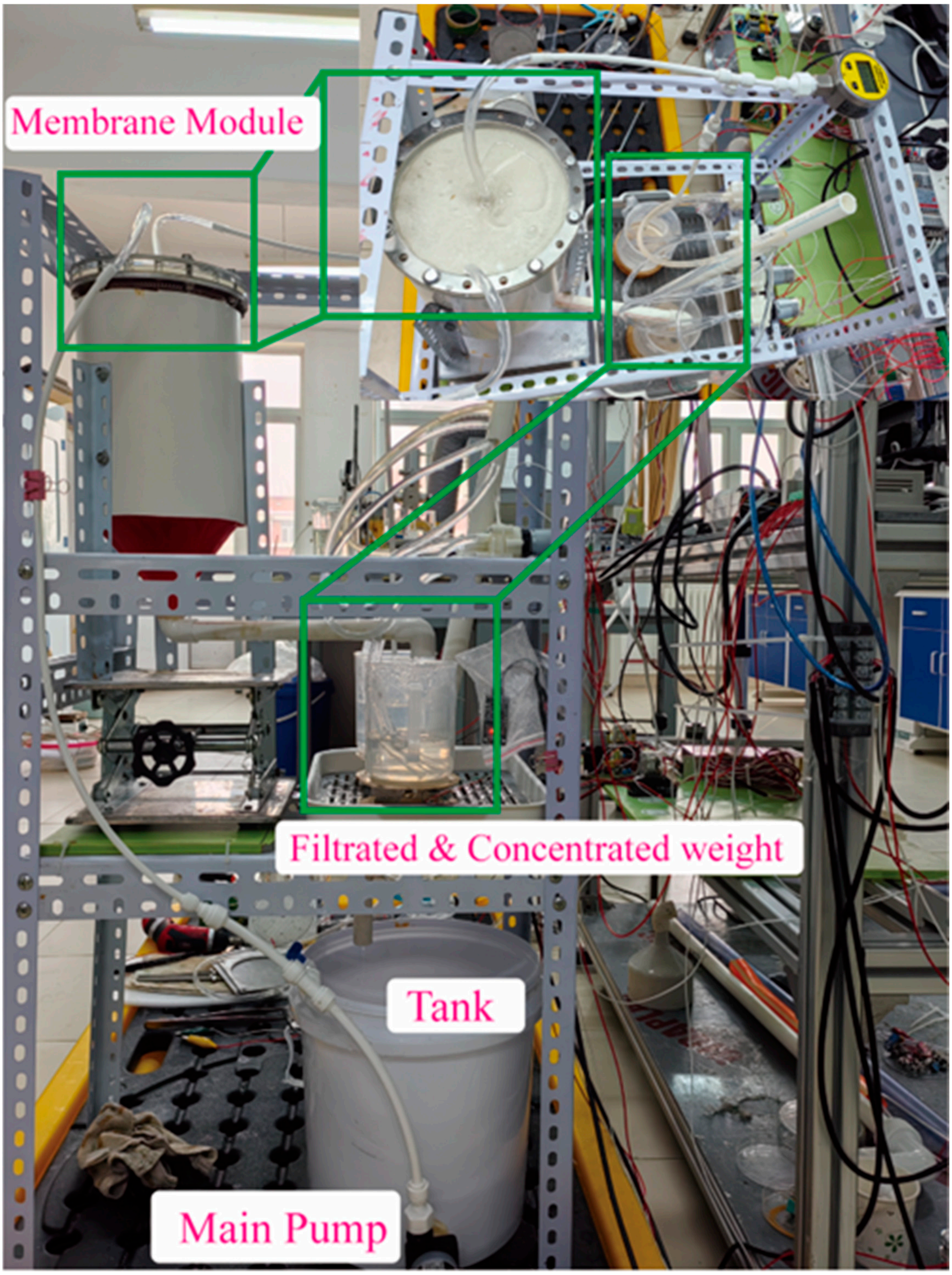

Figure S1. Photograph of the experimental setup.

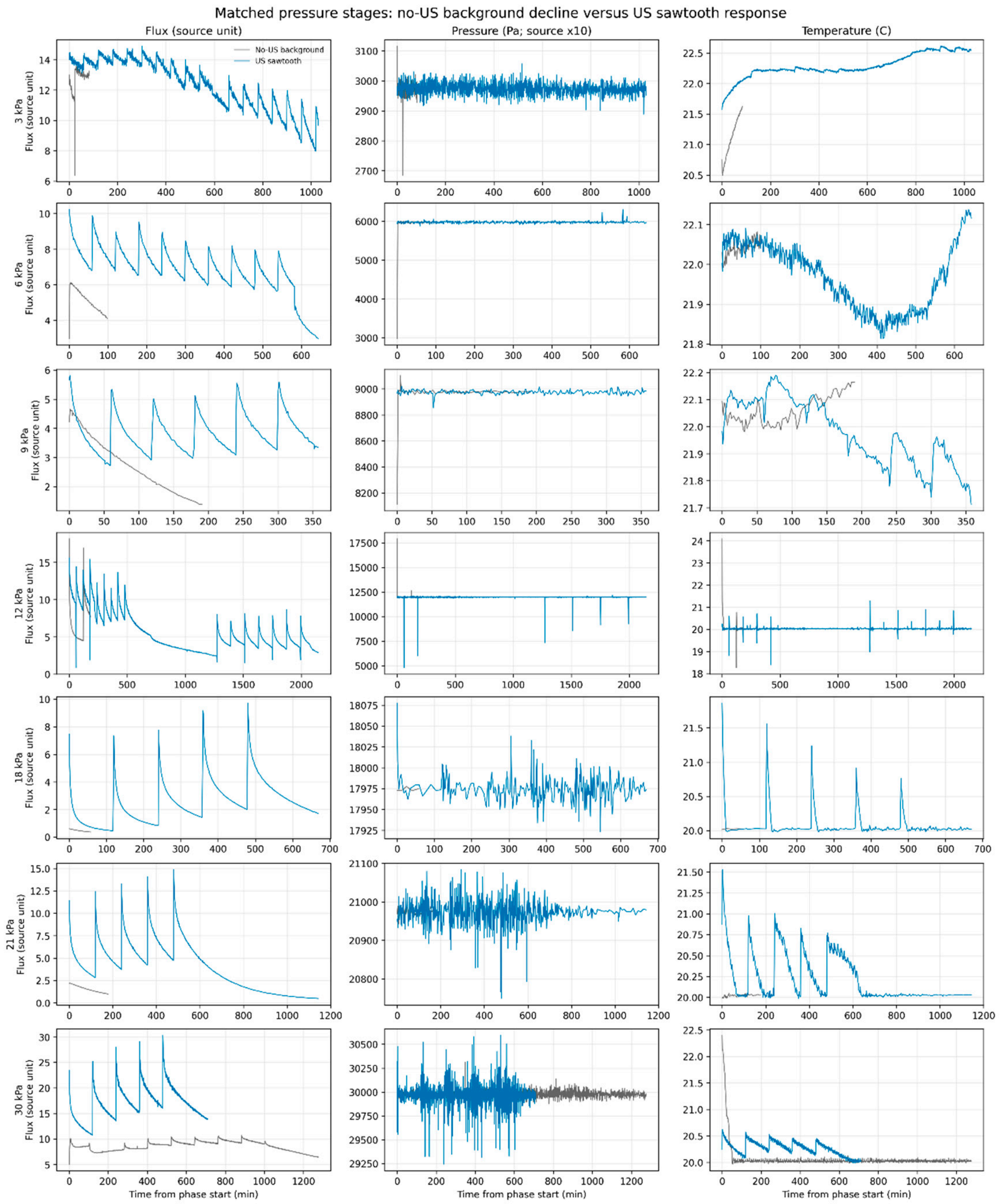

Figure S2. Matched-pressure comparison of baseline flux decline and ultrasound-induced cyclic flux response across 3–30 kPa. Time-resolved flux, transmembrane pressure, and temperature profiles for filtration stages operated at matched pressure levels of 3, 6, 9, 12, 18, 21, and 30 kPa. Gray traces represent baseline filtration without ultrasound (US), whereas blue traces represent filtration under US. At each pressure level, the US condition exhibited periodic sawtooth-like flux changes associated with cyclic ultrasonic action, while the no-US condition showed a comparatively continuous background flux decline. The middle and right columns confirm the corresponding pressure and temperature histories, respectively. Time is referenced to the start of each pressure stage.

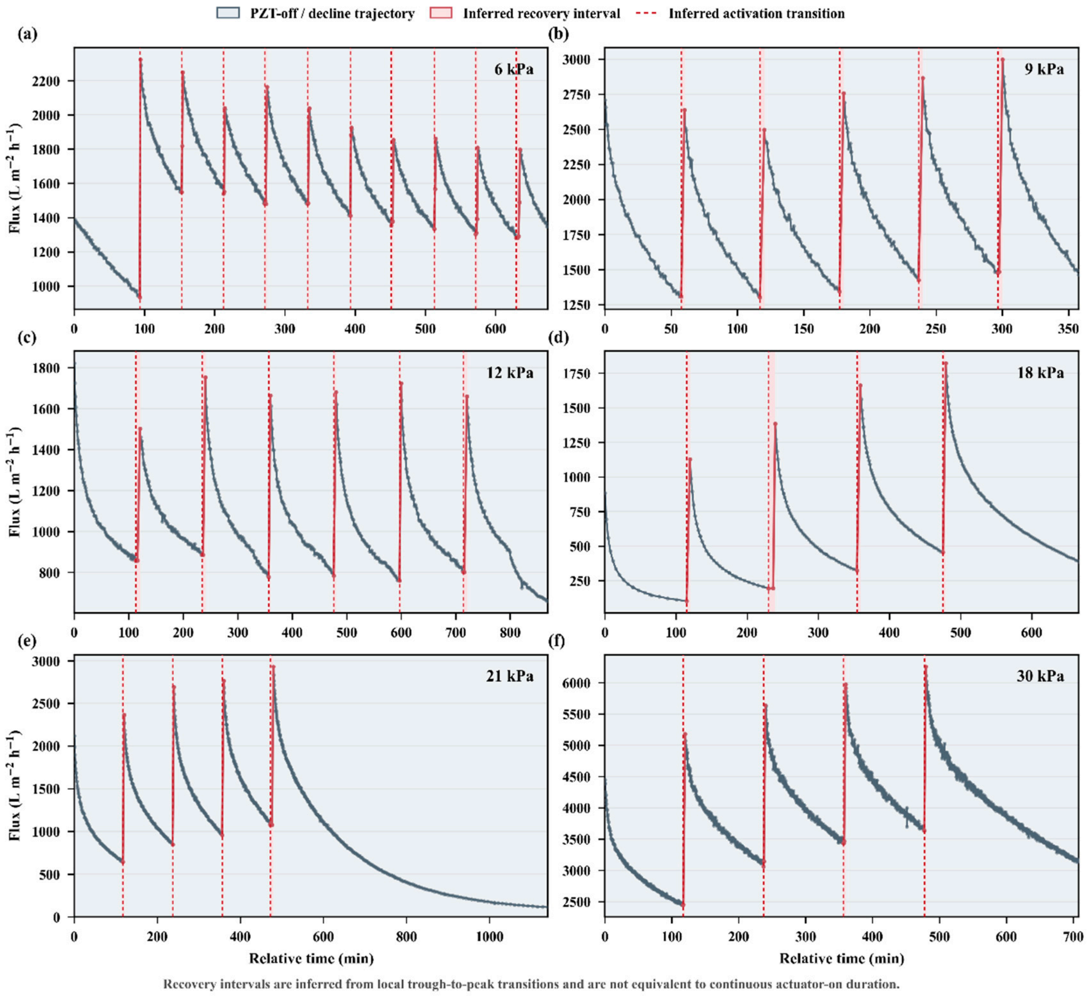

Figure S3. Segmented cyclic flux profiles under six transmembrane pressures. Panels (a-f) show the measured flux records at 6, 9, 12, 18, 21 and 30 kPa, respectively. Grey intervals denote the PZT-off or declining trajectory. Within each candidate recovery segment, the local trough-to-subsequent-peak interval was identified from the largest positive adjacent flux transition and is shown in rose. Red dashed lines mark the inferred onset of these recovery transitions. The inferred intervals are not equivalent to the recorded actuator-on duration and must not be interpreted as continuous PZT-on periods. Flux is reported in L m−2 h−1. The six pressure stages were collected chronologically from two membrane histories and are not independent pressure-treatment replicates.

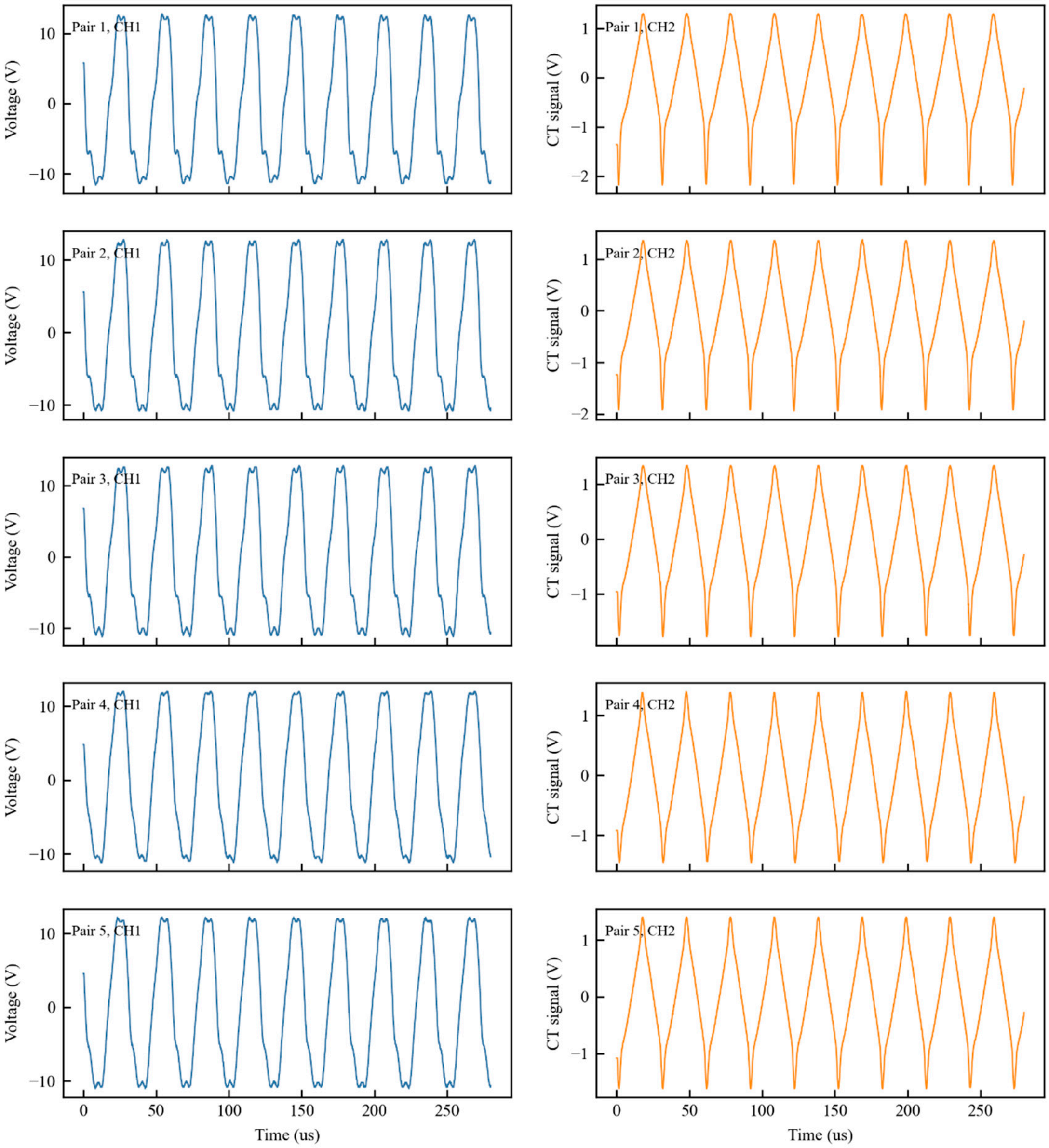

Figure S4. Paired oscilloscope waveforms for PZT electrical characterization. Channel 1 records the PZT terminal voltage through a 100x high-voltage probe, and Channel 2 records the current-transformer burden voltage. The oscilloscope trace was frozen before sequentially exporting the two channels; therefore, each Channel 1/Channel 2 file pair was treated as a synchronized waveform pair.

Section S2. Hermia-cycle descriptors

To evaluate whether Hermia-cycle descriptors reflected the mechanistic parameters of the piezoelectric fouling-cleaning model, a parameter sweep was performed for k_f, k_d and k_{ir}. Each parameter combination generated a 0-600 min cyclic flux curve using the piecewise mechanistic model. Hermia-cycle descriptors were then extracted from each simulated curve following the procedure described in Section 2.5.

Because the variables did not fully satisfy normality assumptions, Spearman rank correlation analysis was used to evaluate monotonic associations between mechanistic inputs and Hermia-cycle descriptors. The

mechanistic variables included k_f, k_d, k_{ir}, k_f+k_{ir}, $k_{ir}/(k_f+k_{ir})$, $R_{ir,final}$ and $R_{ir,final}/R_{total,final}$, depending on the analysis objective. The primary Hermia-cycle descriptors included $\bar{k}_{classic}$, $\bar{k}_{ext}$, J_{final}, $R_{r,final}$, $R_{ir,final}/R_{total,final}$ and the mean flux-loss fraction.

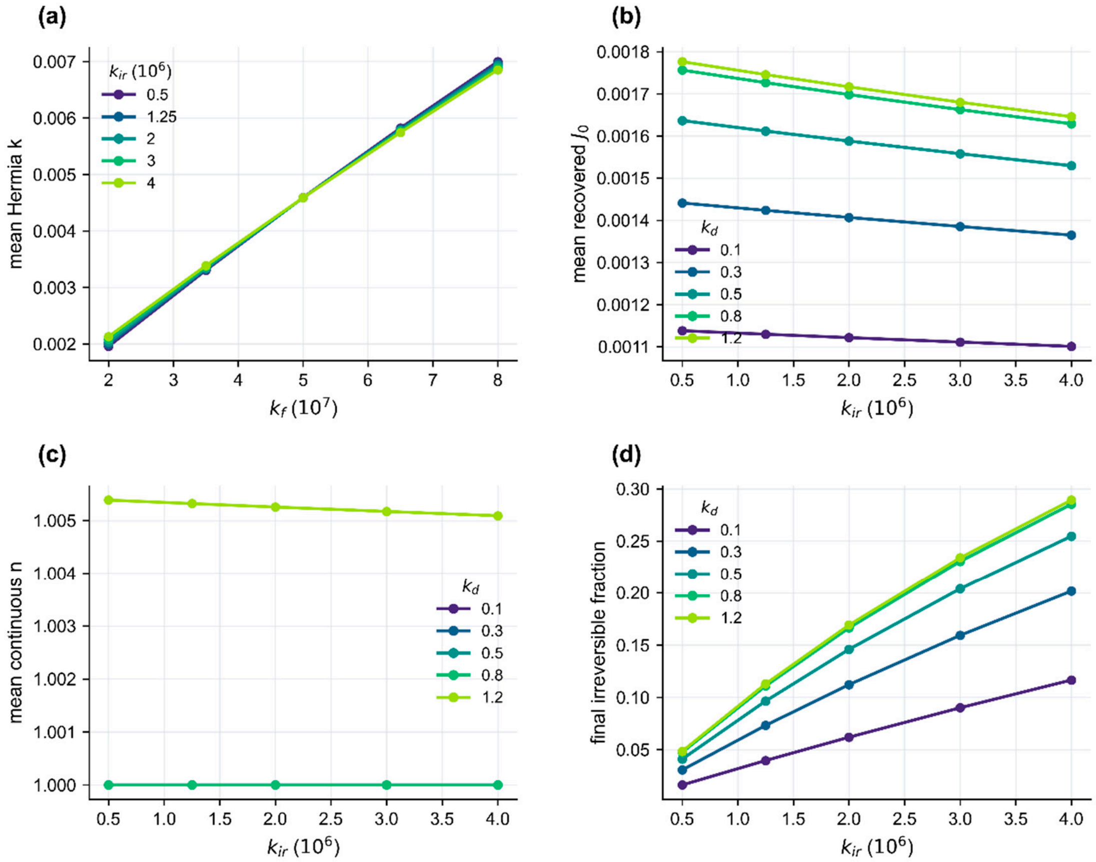

Figure S5. Parameter-sweep simulation of Hermia-cycle descriptors under different mechanistic fouling-cleaning parameters. (a) The mean Hermia kinetic coefficient increased mainly with k_f, whereas different kir levels produced only minor separation. (b) The mean recovered initial flux J0 decreased with increasing kir, while higher k_d improved post-cleaning flux recovery. (c) The mean continuous Hermia mechanism index n remained close to 1.0-1.006, indicating limited variation in the fitted decline-shape descriptor across the tested parameter range. (d) The final irreversible-resistance fraction increased with kir and was also affected by k_d, reflecting the combined influence of irreversible accumulation and piezoelectric cleaning on the final resistance distribution.

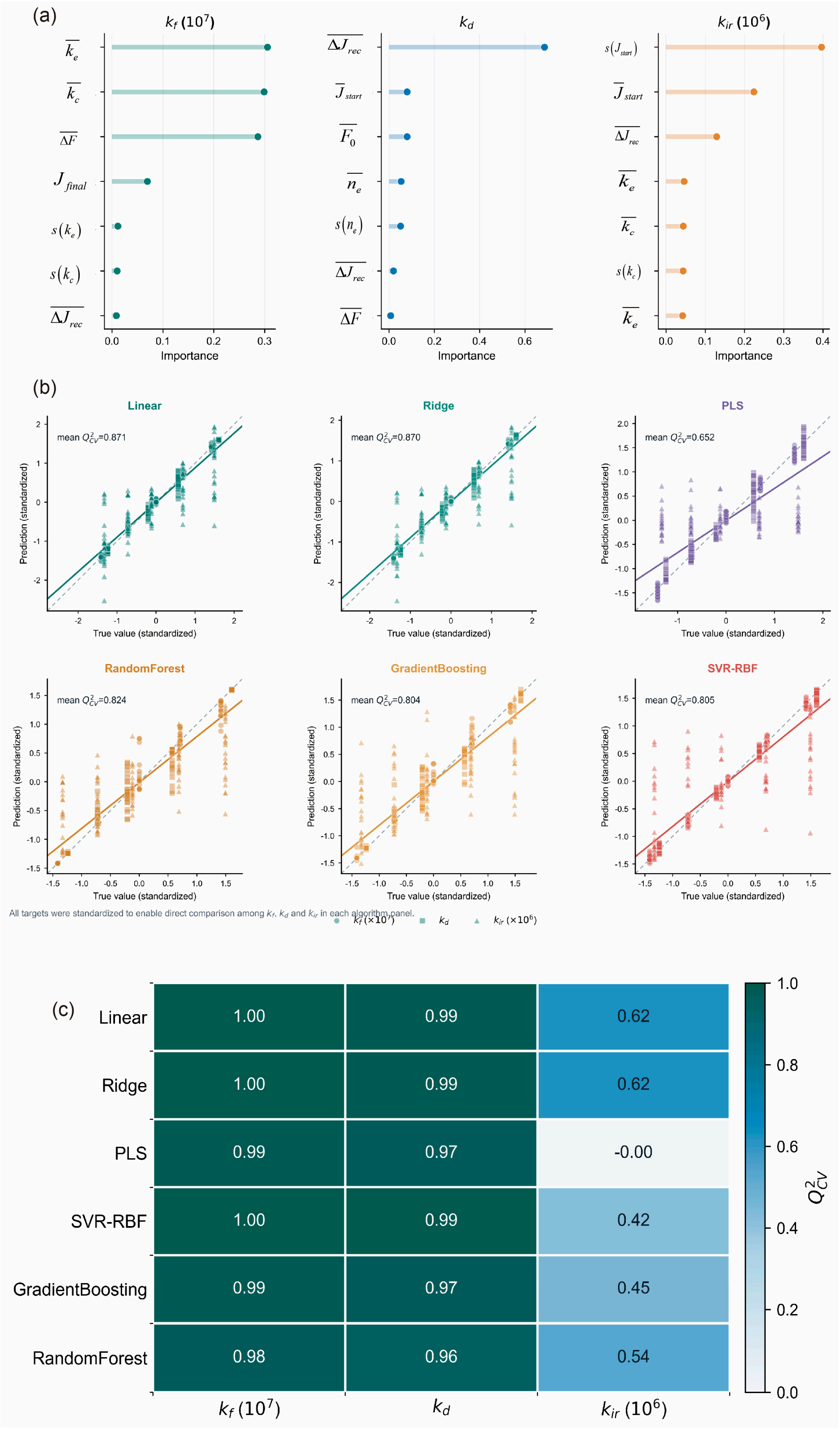

Figure S6. Inverse identification of piezoelectric membrane fouling parameters using Hermia-cycle descriptors. (a) Feature importance of Hermia-cycle descriptors for inverse identification of piezoelectric membrane operating parameters. (b) Standardized scatter visualization of algorithm-level inverse prediction performance. (c) Five-fold cross-validated predictive performance.

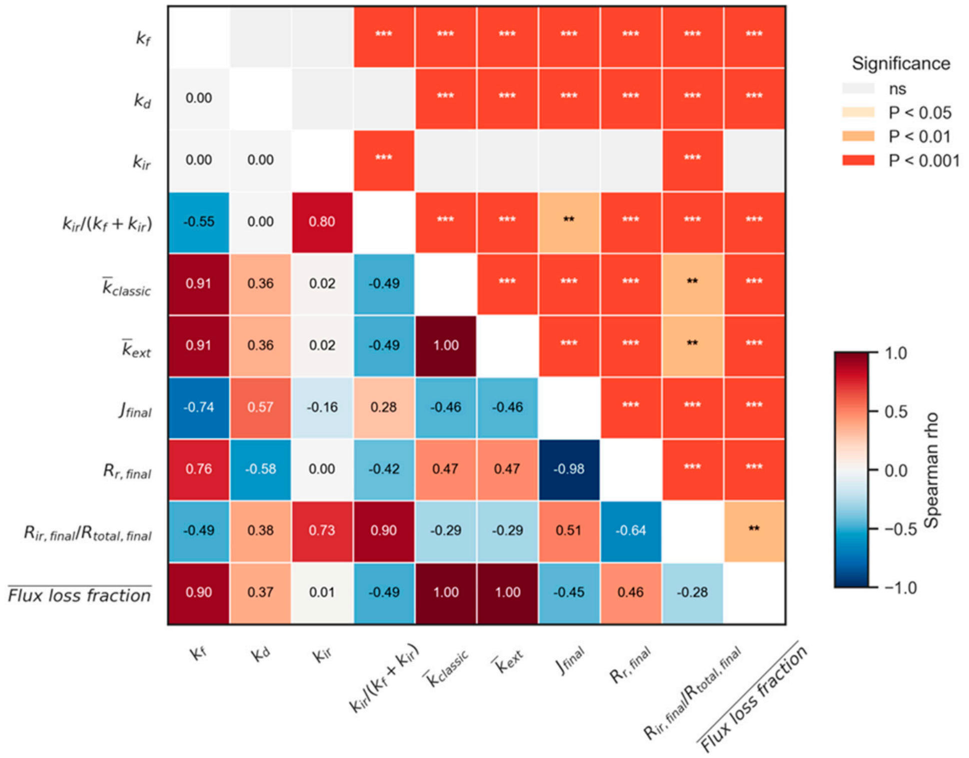

Figure S7. Spearman correlation and significance analysis of mechanistic fouling parameters and Hermia-cycle descriptors. The lower triangle shows Spearman correlation coefficients, with red indicating positive correlations and blue indicating negative correlations. The upper triangle shows significance levels. ns, not significant; *P < 0.05; **P < 0.01; ***P < 0.001.

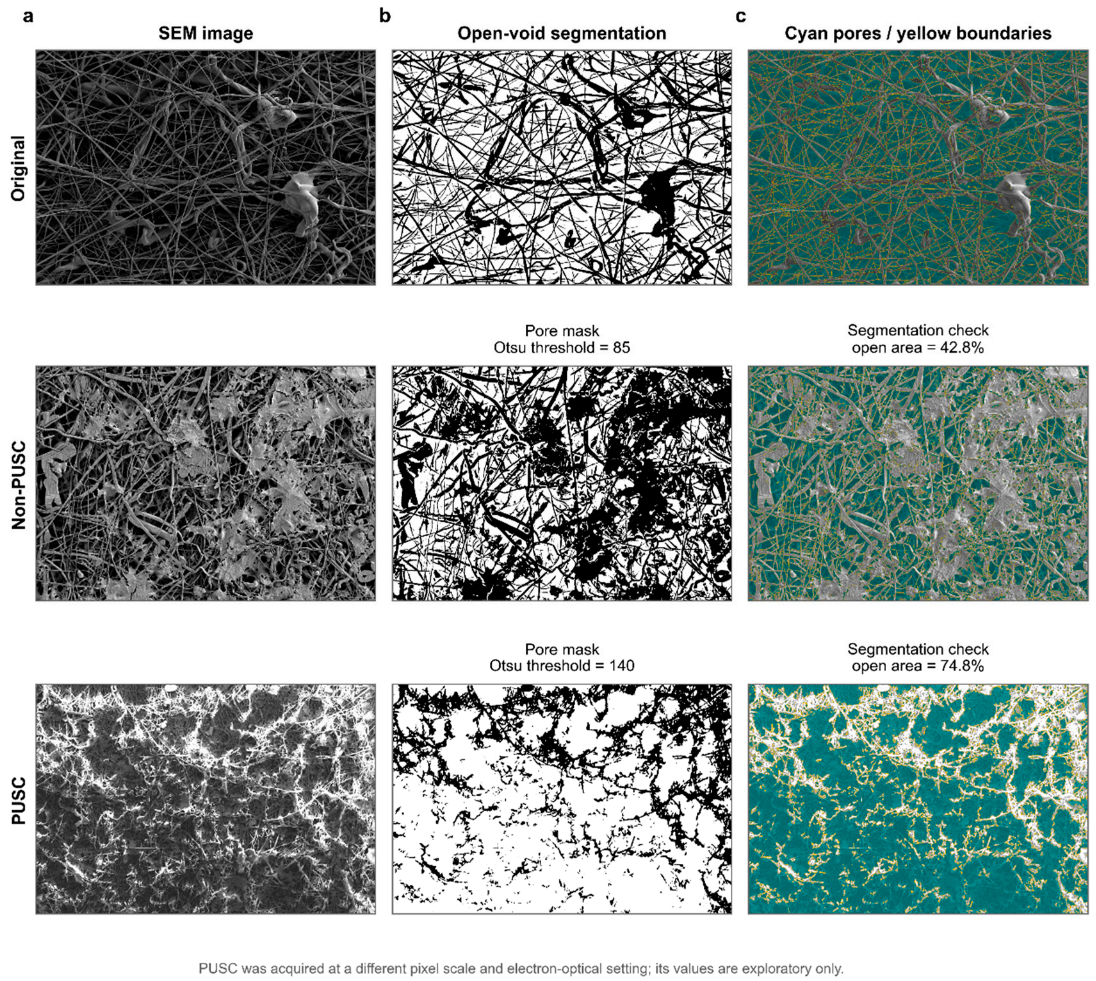

Figure S8. SEM image segmentation workflow for projected open-void quantification. Representative SEM images, binary open-void masks, and segmentation overlays for the Original, Non-PUSC, and PUSC membranes. Cyan regions indicate segmented open pores/voids, and yellow boundaries indicate detected pore boundaries. The PUSC image was acquired at a different pixel scale and electron-optical setting; therefore, its segmentation result was used for exploratory visual comparison rather than direct quantitative comparison with the Original and Non-PUSC groups.

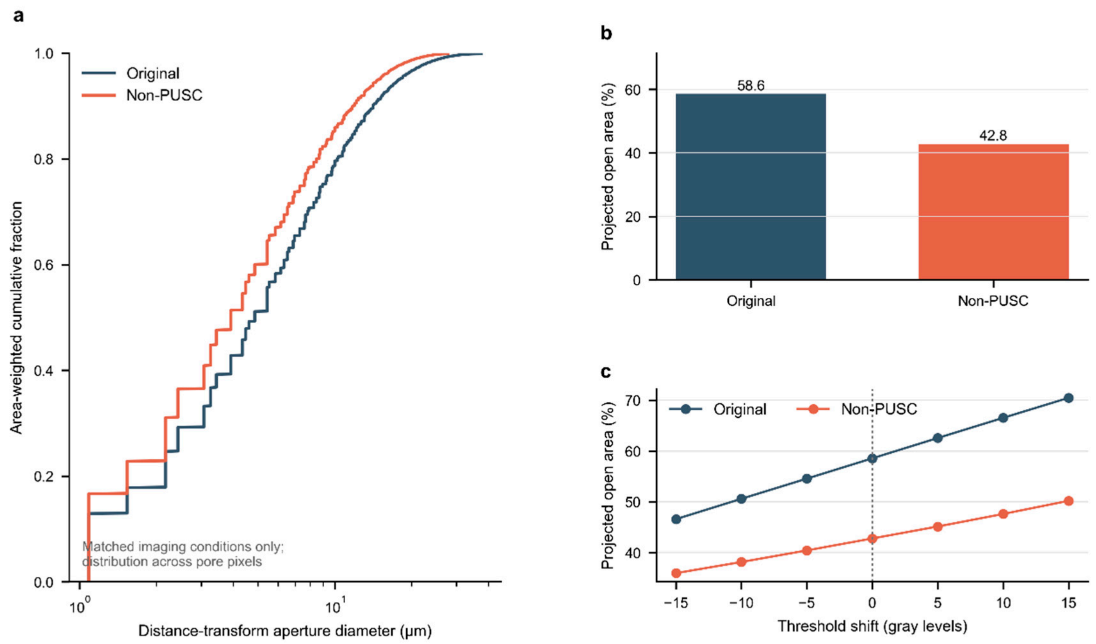

Figure S9. SEM-based projected open-area quantification and threshold-sensitivity analysis. (a) Area-weighted cumulative distribution of distance-transform aperture diameter for the Original and Non-PUSC membranes under matched SEM imaging conditions. (b) Projected open area calculated from the selected segmentation threshold. (c) Sensitivity of projected open-area estimates to threshold shifts. The analysis was restricted to Original and Non-PUSC images acquired under matched imaging conditions; PUSC images were acquired under different imaging settings and were therefore not included in this direct quantitative comparison.

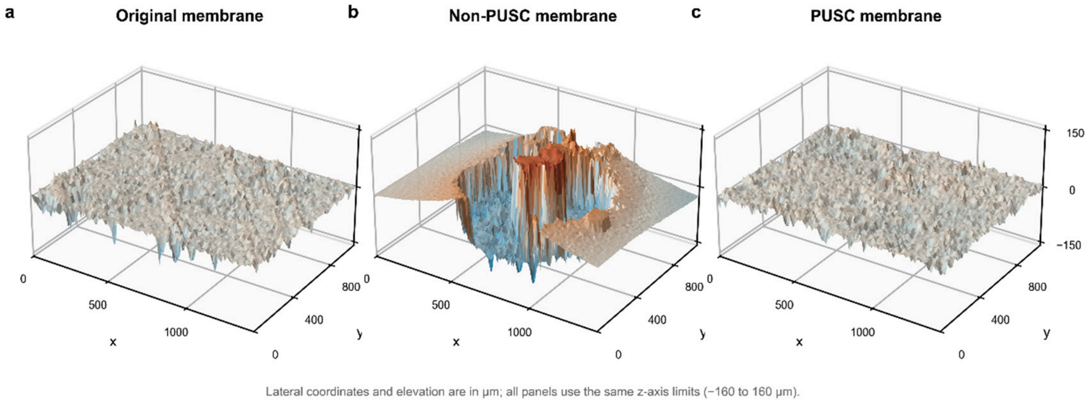

Figure S10. Three-dimensional Keyence surface morphology of the original, Non-PUSC-fouled, and PUSC-cleaned membranes. Three-dimensional surface elevation maps reconstructed from Keyence laser confocal microscopy data for (a) the original membrane, (b) the Non-PUSC-fouled membrane, and (c) the PUSC-cleaned membrane. Lateral coordinates and elevation are shown in μm, and all panels use the same z-axis range to allow direct visual comparison. The Non-PUSC membrane exhibited pronounced vertical heterogeneity caused by fouling-layer accumulation, whereas the PUSC-cleaned membrane showed partial restoration of the original surface morphology.

Section S3. polysaccharide/protein-rich organic fouling matrix analysis

The microstructure and composition of fouling layers were investigated by confocal laser scanning microscopy (CLSM; LSM900, Carl Zeiss AG, Germany). Four fluorescent probes were used to label major polysaccharide/protein-rich organic fouling matrix components: fluorescein isothiocyanate (FITC) for proteins, Concanavalin A conjugated with Alexa Fluor 555 (ConA 555) for alpha-polysaccharides, Calcofluor White for be-ta-polysaccharides and SYTO 63 for nucleic acids [6]. Fluorescent images were ac-quired using multi-channel scanning. Image processing and quantitative analysis were conducted using ImageJ (National Institutes of Health, USA) with the JACoP plugin. Pearson's correlation coefficient (PCC) was used to evaluate pixel-intensity correlation among fluorescence channels, and threshold Manders coefficients were used to de-scribe directional spatial overlap. CLSM was used as a structural and compositional indicator because previous membrane-fouling studies have shown that EPS distribution and bio-cake architecture can control TMP rise and fouling resistance.

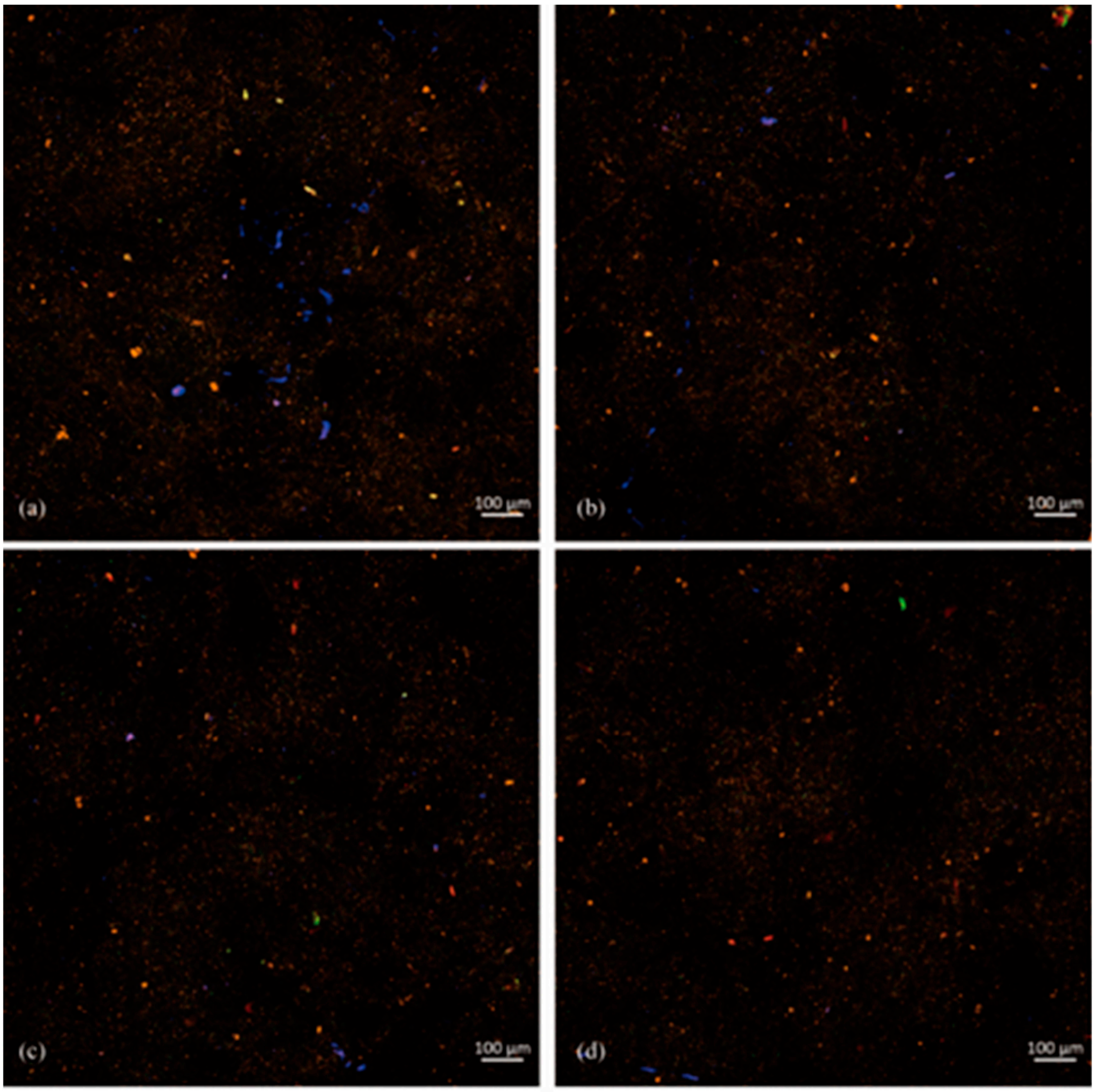

Figure S11. Merged CLSM fluorescence images showing the spatial distributions of α-polysaccharides, β-polysaccharides, proteins, and nucleic-acid-associated components in the pollutant mixture (a), initially fouled membrane (b), non-PUSC membrane (c), and PUSC-cleaned membrane (d).

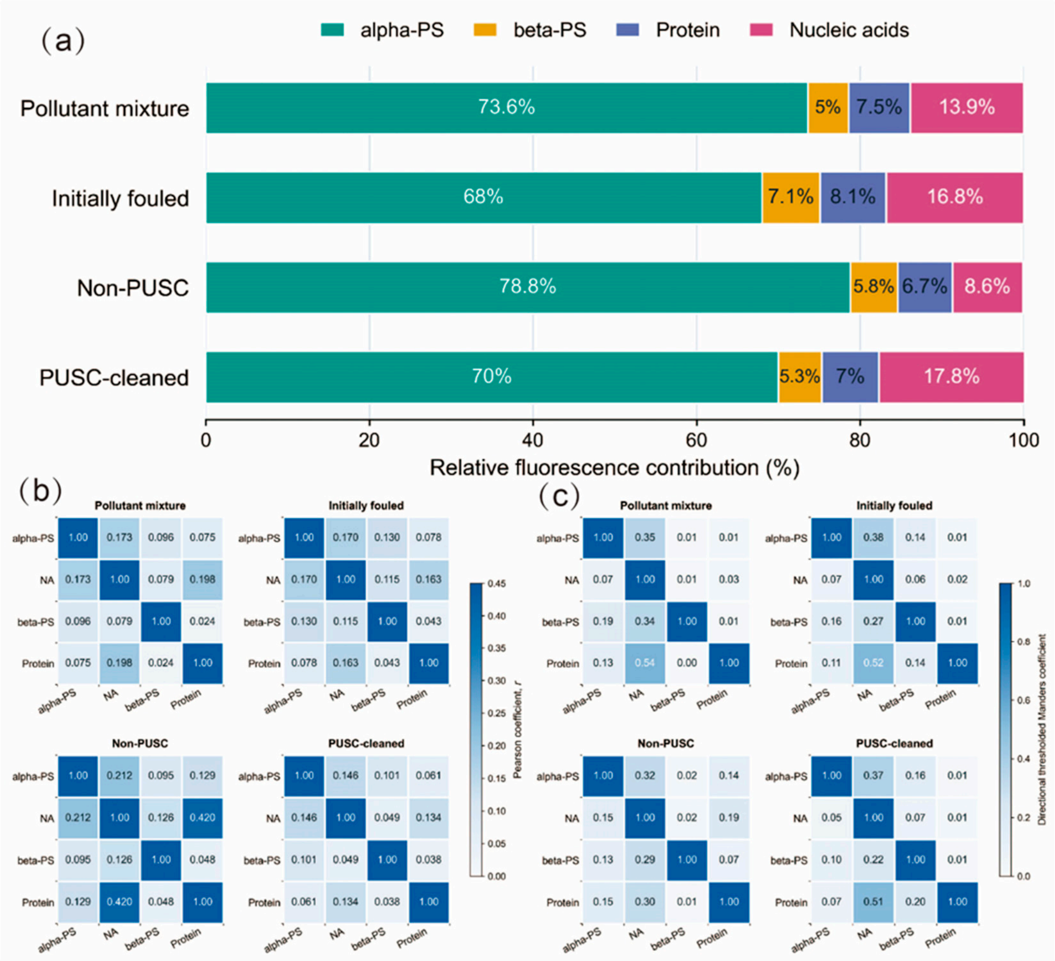

Figure S12. polysaccharide/protein-rich organic fouling matrix composition and spatial colocalization characteristics in the pollutant mixture and membrane samples. (a) Relative fluorescence contributions of α-polysaccharides (α-PS), β-polysaccharides (β-PS), proteins, and nucleic acids. (b) Pearson correlation coefficient matrices describing fluorescence-intensity correlations among polysaccharide/protein-rich organic fouling matrix components. (c) Directional thresholded Manders coefficient matrices describing the spatial overlap of polysaccharide/protein-rich organic fouling matrix components. Samples include the pollutant mixture, initially fouled membrane, non-PUSC membrane, and PUSC membrane.
